# A New Class of Multimerization Selective Inhibitors of HIV-1 Integrase

**DOI:** 10.1371/journal.ppat.1004171

**Published:** 2014-05-29

**Authors:** Amit Sharma, Alison Slaughter, Nivedita Jena, Lei Feng, Jacques J. Kessl, Hind J. Fadel, Nirav Malani, Frances Male, Li Wu, Eric Poeschla, Frederic D. Bushman, James R. Fuchs, Mamuka Kvaratskhelia

**Affiliations:** 1 Center for Retrovirus Research and College of Pharmacy, The Ohio State University, Columbus, Ohio, United States of America; 2 Division of Medicinal Chemistry and Pharmacognosy, College of Pharmacy, The Ohio State University, Columbus, Ohio, United States of America; 3 Department of Molecular Medicine & Division of Infectious Diseases, Mayo Clinic College of Medicine, Rochester, Minnesota, United States of America; 4 Perelman School of Medicine, Department of Microbiology, University of Pennsylvania, Philadelphia, Pennsylvania, United States of America; 5 Center for Retrovirus Research and Department of Veterinary Biosciences, The Ohio State University, Columbus, Ohio, United States of America; Duke University Medical Center, United States of America

## Abstract

The quinoline-based allosteric HIV-1 integrase (IN) inhibitors (ALLINIs) are promising candidates for clinically useful antiviral agents. Studies using these compounds have highlighted the role of IN in both early and late stages of virus replication. However, dissecting the exact mechanism of action of the quinoline-based ALLINIs has been complicated by the multifunctional nature of these inhibitors because they both inhibit IN binding with its cofactor LEDGF/p75 and promote aberrant IN multimerization with similar potencies *in vitro*. Here we report design of small molecules that allowed us to probe the role of HIV-1 IN multimerization independently from IN-LEDGF/p75 interactions in infected cells. We altered the rigid quinoline moiety in ALLINIs and designed pyridine-based molecules with a rotatable single bond to allow these compounds to bridge between interacting IN subunits optimally and promote oligomerization. The most potent pyridine-based inhibitor, KF116, potently (EC_50_ of 0.024 µM) blocked HIV-1 replication by inducing aberrant IN multimerization in virus particles, whereas it was not effective when added to target cells. Furthermore, KF116 inhibited the HIV-1 IN variant with the A128T substitution, which confers resistance to the majority of quinoline-based ALLINIs. A genome-wide HIV-1 integration site analysis demonstrated that addition of KF116 to target or producer cells did not affect LEDGF/p75-dependent HIV-1 integration in host chromosomes, indicating that this compound is not detectably inhibiting IN-LEDGF/p75 binding. These findings delineate the significance of correctly ordered IN structure for HIV-1 particle morphogenesis and demonstrate feasibility of exploiting IN multimerization as a therapeutic target. Furthermore, pyridine-based compounds present a novel class of multimerization selective IN inhibitors as investigational probes for HIV-1 molecular biology.

## Author Summary

The administration of highly active-antiretroviral therapy (HAART) has changed what was once a terminal disease into a manageable chronic infection. The success of HAART is manifested by reduced mortality and morbidity of HIV-1 infected patients. However, evolution of HIV-1 strains resistant to current therapies is a major clinical problem in the fight against AIDS. Therefore, new inhibitors with novel mechanisms of action are needed. One such mechanism is to target multimerization of HIV-1 integrase. In the present study, we report the design of pyridine-based small molecules that contain a rotatable single bond to allow optimal bridging between interacting integrase subunits. As a result, pyridine-based compounds stabilized interacting IN subunits and promoted aberrant, higher order integrase multimerization. The most potent compound, KF116, potently inhibited HIV-1 replication by interfering with proper maturation of HIV-1 particles, whereas KF116 at therapeutically relevant (submicromolar) concentrations had no detectable effects on LEDGF/p75 mediated HIV-1 integration. Our findings highlight HIV-1 integrase multimerization as a plausible therapeutic target and offer a path for designing improved inhibitors for potential clinical use.

## Introduction

HIV-1 integrase (IN) is an important therapeutic target as its function is essential for viral replication. A tetramer of IN assembles on the viral DNA ends to form the stable synaptic complex (SSC) and catalyzes two reactions necessary for the integration of linear viral DNA into the host chromatin [1]. IN initially removes a GT dinucleotide from the 3′-terminus of each viral DNA end (3′-processing), and then integrates recessed viral DNA ends into the target DNA in a staggered fashion through concerted transesterification reactions (DNA strand transfer). IN is comprised of three domains: the N-terminal domain (NTD) which contains the Zn-binding motif (HH-CC type), the catalytic core domain (CCD) which contains the DDE catalytic triad that coordinates essential Mg^2+^ ions, and the C-terminal domain (CTD) which contains an SH3-like fold (reviewed in [2]). Each of these domains contributes to protein multimerization [3]–[6].

In infected cells, IN assembles with a number of viral and cellular proteins to form a larger nucleoprotein complex termed the preintegration complex (PIC). Cellular chromatin associated protein LEDGF/p75 is a key binding partner of HIV-1 IN and promotes effective tethering of PICs to active genes during integration [7]–[13]. The C-terminal domain of LEDGF/p75, termed the Integrase Binding Domain (IBD), stabilizes the IN tetramer by engaging the IN CCD dimer interface and the NTD of another dimer [13]–[16]. The N-terminal portion of LEDGF/p75, which contains a PWWP domain, nuclear localization signal, AT hooks and highly charged regions, associates with chromatin through engaging both nucleosomal DNA and the trimethylated H3 tail (H3K36me3) [17]–[19], which is an epigenetic marker for active genes and positively correlates with HIV-1 integration sites [20].

The catalytic function of HIV-1 IN has been exploited as a therapeutic target. Three HIV-1 IN inhibitors, raltegravir (RAL), elvitegravir (EVG) and dolutegravir (DTG), are currently in clinical use [21], [22]. These inhibitors bind at the enzyme active site and inhibit DNA strand transfer (termed IN strand transfer inhibitors or INSTIs). HIV-1 IN mutations that alter amino-acids near the INSTI binding site can confer resistance to first generation INSTIs RAL and EVG, and have emerged in patients receiving treatment [23]–[25]. Second generation INSTI DTG retains significant activity against many RAL and EVG resistance mutations and appears to have a higher genetic barrier to resistance. However, IN mutations that confer low-level resistance to DTG have been recently reported [26]. Thus the development of small molecules that impair IN function with different mechanisms of action while retaining potency against INSTI resistant viruses is an important goal.

One such mechanism is to target IN multimerization. We have reported studies demonstrating a highly dynamic nature of HIV-1 IN subunit-subunit interactions and that such flexibility is important for the assembly of the SSC [16], [27]. Various ligands that bind at the IN CCD dimer interface can bridge interacting IN subunits and promote aberrant IN multimerization [28]–[31]. The term “aberrant IN multimerization” is used here to refer to the formation of catalytically inactive IN oligomers that differ from the correctly assembled catalytically active tetramer found in the SSC. A significant advantage of stabilizing rather than destabilizing interacting subunits is that small molecule inhibitors do not have to overcome the energy barriers associated with the formation of large protein-protein interfaces. In proof-of-concept studies [30] a small molecule inhibited IN activity *in vitro* by binding at the IN CCD dimer interface, stabilizing the interacting subunits and promoting aberrant IN multimerization.

Recently, quinoline-based allosteric IN inhibitors (referred to here as ALLINIs) have been reported that target the clinically unexploited LEDGF/p75 binding site at the IN CCD dimer interface and potently inhibit HIV-1 replication in cell culture [32]–[38]. An ALLINI carboxylic acid hydrogen bonds with one IN subunit, potentially mimicing an interaction with LEDGF/p75. The quinoline-ring, another key structural feature of ALLINIs, engages another subunit of IN through hydrophobic interactions [32], [33], [35]. The initial report [32] suggested that these compounds selectively impair the IN-LEDGF/p75 interaction. However, follow up studies [33]–[35], [38] have demonstrated that the quinoline-based ALLINIs inhibit both IN-LEDGF/p75 binding and LEDGF/p75-independent activities with similar IC_50_ values *in vitro*. The underlying mechanism for inhibiting the catalytic function of IN has been shown to be promoting aberrant IN multimerization [33]–[35]. This multimodal mechanism of action of ALLINIs concordantly resulted in cooperative inhibition of HIV-1 replication in cell culture [33], [38]. Selection of viral strains emerging under ALLINI pressure revealed changes near the inhibitor binding site on IN. In particular, A128T substitution is most common and has been shown to confer marked resistance to the majority of ALLINIs [32], [35], [39], [40].

Studies on the antiviral mechanism of action of ALLINIs have unexpectedly revealed that these compounds inhibit both early and late stages of HIV-1 replication [32]–[38], [41], [42]. In fact, different ALLINIs were 3- to 40-fold more potent when added to producer cells versus target cells [41], [42]. In target cells ALLINIs did not affect reverse transcription but did impair HIV-1 integration. The treatment of producer cells with ALLINIs resulted in virions with conical cores that were devoid of electron dense material, likely corresponding to ribonucleic acid-protein complexes (RNPs). Strikingly, RNPs were mislocalized between the core and viral membrane [38], [41], [42]. The viral particles produced in the presence of ALLINIs entered target cells normally but were defective for subsequent reverse transcription in target cells. A single substitution in HIV-1 IN at the inhibitor binding site which confers resistance to quinoline-based ALLINIs was able to overcome these late stage affects [38], [40], [41]. Furthermore, ΔIN viruses transcomplemented with wild type Vpr-IN were fully susceptible to ALLINI inhibition, whereas Vpr-IN containing a substitution in IN at the inhibitor binding pocket exhibited marked resistance [38]. Taken together, these studies demonstrate that ALLINIs target IN during the late stages of HIV-1 replication and compromise the assembly of electron dense cores. However, dissecting the exact mechanism of action of the quinoline-based ALLINIs has been complicated because they both inhibit IN-LEDGF/p75 binding and promote aberrant IN multimerization with similar potency *in vitro* [33]–[35].

Here we report design of small molecules that allowed us to probe the role of HIV-1 IN multimerization independently from IN-LEDGF/p75 interactions. We named these selective multimeric IN inhibitors or MINIs. For this, we have exploited the available X-ray crystal structures of quinoline-based ALLINIs bound to the HIV-1 IN CCD dimer to modify the existing scaffold. In particular, we have altered the quinoline moiety and made a series of modifications to enhance potency specifically for modulating IN multimerization without significantly affecting IN-LEDGF/p75 binding. These studies have resulted in a new class of pyridine-based MINIs, which potently modulate IN multimerization but are not effective inhibitors of IN-LEDGF/p75 binding. Using these new compounds as investigational probes we have delineated the significance of ordered IN structure for the formation of correctly assembled virus particles. Furthermore, MINIs inhibited the HIV-1 variant with the A128T IN substitution, which confers resistance to the majority of ALLINIs. These findings indicate the feasibility of our rational modification approaches to improving IN inhibitors and present a novel class of multimerization selective IN inhibitors as investigational probes.

## Results

### Rational design and structure-activity relationship studies of MINIs

**Figure 1:**
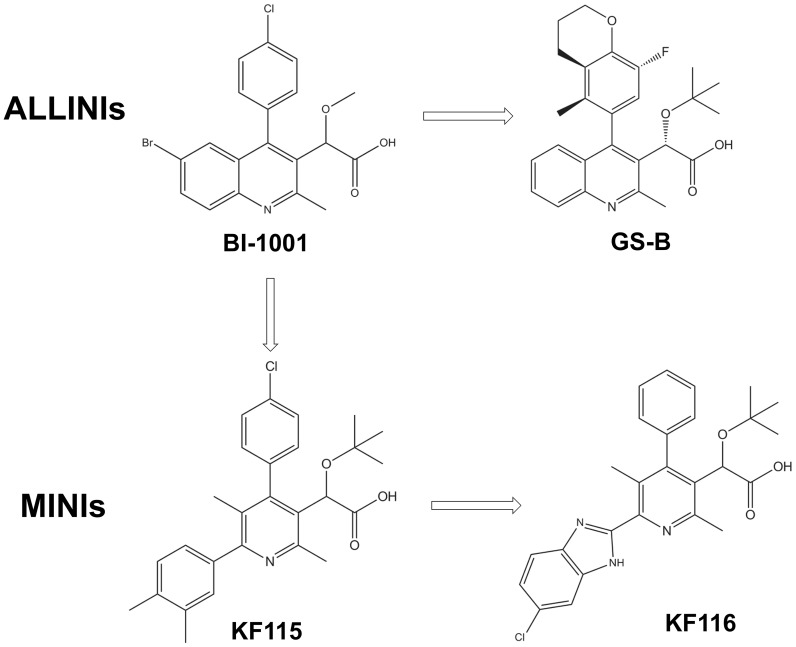
Chemical structures of ALLINIs and MINIs.

The archetypal ALLINI, BI-1001 (Figure 1) is composed of four structural elements: quinoline ring, substituted benzene ring, carboxylic acid and a methoxy group [33], [43]. Optimization of the BI-1001 scaffold has led to the development of GS-B, one of the most potent ALLINIs reported to date [35]. As shown in Figure 1, GS-B possesses an optimized benzene ring and a sterically bulky tert-butoxy ether moiety, which together are responsible for the enhanced antiviral activity (IC_50_ of ∼5.8 µM for BI-1001 vs IC_50_ of ∼0.026 µM for GS-B) [33], [41]. During the course of optimization [35], however, two of the four key structural elements, the quinoline ring and carboxylic acid were not modified. In the present study, we have modified the quinoline ring as explained below.

**Figure 2:**
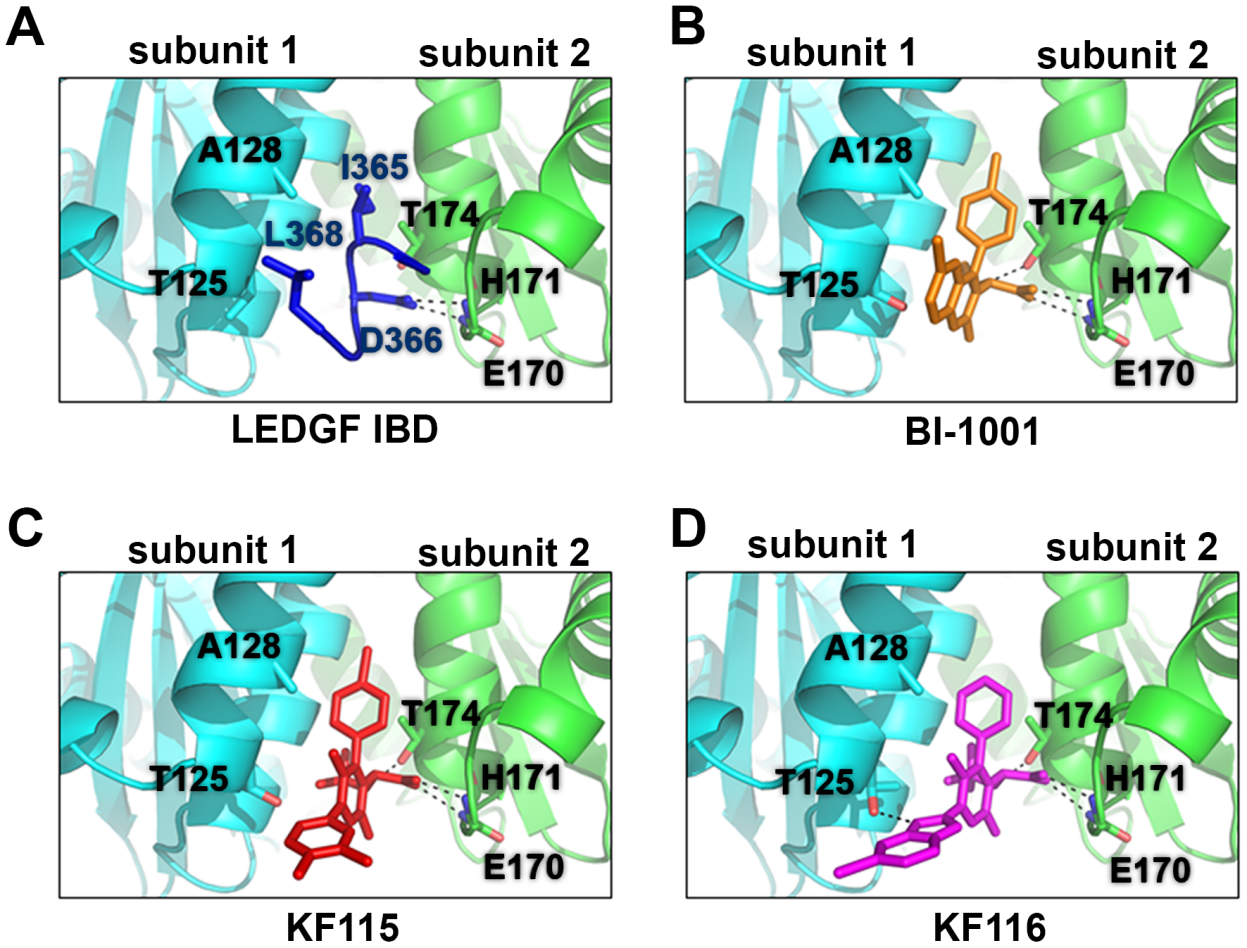
Crystal structures of LEDGF/IBD (A), BI-1001 (B), KF115 (C), and KF116 (D) bound to HIV-1 IN CCD. The IN subunit 1 and 2 are colored in cyan and green, respectively. LEDGF/IBD loop (amino acids 365–368) is shown in dark blue. BI-1001 is shown in orange. KF115 is shown in red. KF116 is shown in magenta. The hydrogen bonds between the IN subunit and the LEDGF/IBD or the indicated inhibitors are shown by black dashed lines. Side chains of HIV-1 IN residues A128T and T125 in subunit 1, and E170, H171 and T174 in subunit 2 are shown.

A comparison of the available crystal structures of BI-1001 and LEDGF/IBD bound to HIV-1 IN CCD dimers [13], [33] shows that BI-1001 binds at the LEDGF/p75 binding site and bridges between two IN subunits (compare Figure 2A and 2B), providing a candidate structural explanation for the multimodal mechanism of action of ALLINIs. The compounds both promote aberrant IN multimerization and inhibit IN-LEDGF/p75 binding with very similar IC_50_ values *in vitro* [33]–[35]. Both LEDGF/p75 and BI-1001 interactions with the IN CCD dimer are anchored by hydrogen bonding interactions with subunit 2 through the main chain nitrogens of IN residues Glu-170 and His-171. An additional key hydrogen bond is also formed, however, between the ether oxygen of BI-1001 and Thr-174 of subunit 2 ([33], [40], also see Figure 2B).

In contrast with subunit 2, interactions with subunit 1 are primarily hydrophobic in nature as observed with LEDGF/p75 Ile-365 and Leu-368 (Figure 2A) or the substituted benzene ring of BI-1001 (Figure 2B). The rigid and planar quinoline scaffold of ALLINIs significantly limits its interactions with subunit 1, although its projection towards Ala-128 of subunit 1 ultimately results in marked resistance to the majority of ALLINIs through emergence of A128T substitution [39].

To enhance the interactions of the inhibitor with subunit 1, we have developed pyridine-based compounds, KF115 and KF116 (Figure 1), through dissociation of the fused quinoline moiety (Figure S1 and Text S1). We hypothesized that the rotatable bond connecting the pyridine ring with the benzene ring in KF115 or with the benzimidazole ring in KF116 would allow these structural motifs to adopt suitable orientations to optimally engage subunit 1.

To test our hypothesis we have solved the crystal structures of KF115 and KF116 bound to HIV-1 IN CCD dimer (Figure 2C and 2D). The results in Figure 2 show that KF115 and KF116 maintain hydrogen bonding with Glu-170 and His-171, and Thr-174 of subunit 2. However, interactions of KF115 and KF116 with subunit 1 significantly deviate from the ALLINIs and LEDGF/p75. In particular, the following two principal differences are noteworthy.

- Pyridine-based KF115 and KF116 establish more extensive interactions with subunit 1 compared with their quinoline-based ALLINI counterparts or LEDGF/p75. KF115 has significantly better overall hydrophobic interactions with subunit 1 compared with ALLINIs or LEDGF/p75. Unlike the rigid quinoline scaffold in ALLINIs, the dimethylated benzene ring of KF115 takes advantage of the more flexible single bond and adopts a suitable conformation to obtain maximal hydrophobic interactions of the aryl unit with IN subunit 1. As anticipated, the observed perpendicular conformation of the biaryl system responsible for projecting the aromatic ring toward subunit 1 is enforced by the presence of the methyl substituents on the central pyridine ring. Moreover, the benzimidazole ring of KF116, which adopts a similar conformation, engages subunit 1 through an additional hydrogen bonding interaction with the side chain of Thr-125.The additional interactions of KF115 and KF116 with subunit 1 were expected to significantly enhance their ability to bridge between two IN subunits and accordingly enhance aberrant IN multimerization.
- Interactions of the ALLINI quinoline group with IN subunit 1 closely mimic LEDGF/p75 Leu-368, whereas KF115 and KF116 lack this quinoline moiety. Accordingly, we hypothesized that these changes would reduce the specificity of KF115 and KF116 for affecting IN-LEDGF/p75 binding compared with their quinoline-based ALLINI counterparts.

To test our hypotheses and dissect the mode of action of KF115 and KF116, we have evaluated their IC_50_ values for promoting IN multimerization and inhibiting the IN-LEDGF/p75 interaction *in vitro*. The data in Table 1 and Figure S2 show that KF115 and KF116 were ∼50- and ∼60-fold more selective for modulating IN multimerization compared with IN-LEDGF/p75 binding (selectivity ratio defined as IC_50_ for inhibiting IN-LEDGF/p75 binding divided by EC_50_ for aberrant IN multimerization, Table 1). In control experiments, archetypal ALLINI BI-1001 exhibited comparable potency for inhibiting IN-LEDGF/p75 binding compared with aberrant IN multimerization (0.2-fold selectivity ratio). Of note, all multifunctional ALLINIs reported to date have been shown to exhibit comparable potencies for inhibiting IN-LEDGF/p75 binding and promoting aberrant IN multimerization ([33]–[35], [38], [40], also see Table 1).

**Table 1:** Activities of HIV-1 IN inhibitors.

compound	EC_50_ for aberrant IN multimerization, µM	IC_50_ for IN-LEDGF/75 binding, µM	Selectivity ratio, IC_50_ IN-LEDGF/EC_50_ aberrant IN multimerization	Antiviral activity (IC_50_), µM	Cytotoxicty (CC_50_), µM	Selectivity Index, CC_50_/IC_50_
BI-1001	4.9±0.3^a^	1.0±0.1^a^	0.2	5.8±0.1^a^	>100	>17
KF115	0.274±0.025	13.0±1.5	47.4	0.121±0.004	>100	>826
KF116	0.086±0.006	5.03±0.36	58.5	0.024±0.003	>100	>4167

Data for IC_50_ and EC_50_ are given as the mean ± SD from at least three independent experiments.

CC_50_ values of >100 µM indicates that the respective inhibitors were not cytotoxic at the tested concentrations upto 100 µM.

a BI-1001 EC_50_ value and the assay method have been described elsewhere [33].

We next investigated whether the high degree of selectivity of KF115 and KF116 for modulating IN multimerization could affect the antiviral potency of these compounds. The results in Table 1 show that KF115 and KF116 inhibited HIV-1 replication in infected cells with an IC_50_ value of ∼0.121 µM and ∼0.024 µM, respectively, whereas no cytotoxicity was detected with 100 µM (the highest concentration tested) of either of these compounds. Accordingly, the selectivity indexes (defined as CC_50_ for cytotoxicity by IC_50_ for antiviral activity) for KF115 and KF116 are >826 and >4,000, respectively (Table 1). Since KF115 and KF116 IC_50_ values for antiviral activities in infected cells correlate closely with EC_50_ for promoting aberrant IN multimerization but not with IC_50_ for inhibiting the IN-LEDGF/p75 binding, we conclude that the antiviral potencies of KF115 and KF116 are determined primarily through promoting aberrant IN multimerization. Accordingly, we refer to KF115 and KF116 as multimeric IN inhibitors or MINIs. KF116 was chosen for further mechnaistic studies due to its more potent antiviral activities.

IN residue Thr-125, which forms a hydrogen bond with KF116 (Figure 2D), predominates in clade B and is present in HIV-1_NL4-3_. However, the majority of clade C strains contain Ala at this position. Therefore, we examined the antiviral activities of KF116 with respect to HIV-1 clade C using the infectious molecular clone pMJ4, which contains IN residue Ala125 [44]. KF116 inhibited HIV-1_MJ4_ replication with an IC_50_ of 0.127±0.01 µM. The observed differences in KF116 IC_50_ values for HIV-1_NL4-3_ (∼0.024 µM) versus HIV-1_MJ4_ (∼0.127 µM) could be due to the presence of Thr or Ala respectively at IN residue 125. This would be consistent with the crystallographic results (Figure 2D) where Thr125 was found to hydrogen bond with KF116, whereas Ala125 would lack the ability to form a hydrogen bond with KF116, resulting in decreased potency. Thus the strategy of linking the pyridine and benzimidazole rings through a rotatable bond allows the inhibitor to engage both interacting subunits even with residue variations at position 125, thereby preserving activity against subtype C.

### Evolution of HIV-1 variants in the presence of KF116

The A128T IN substitution is the most prevalent mutation conferring resistance to most quinoline-based ALLINIs, including BI-1001 [32], [35], [38]–[40]. Structural and biochemical analyses have elucidated the underlying mechanism for HIV-1 A128T IN resistance to ALLINIs [40]. The biochemical studies showed that the A128T substitution has evolved to overcome BI-1001 induced IN multimerization rather than the inhibition of IN-LEDGF/p75 binding. In particular, our published crystal structures of BI-1001 bound to WT and A128T IN CCDs revealed that the A128T substitution affected the positioning of the quinoline group through the bulkier and polar threonine exerting a steric effect and electronic repulsion to BI-1001 ([40], also see Figure S3A). Since KF116 lacks the quinoline moiety, we hypothesized that this inhibitor could escape the effects of threonine at position 128. Indeed, our crystal structures have revealed very similar binding of KF116 to WT and A128T IN CCDs (Figure S3B). Moreover, KF116 potently promoted aberrant IN multimerization of A128T IN *in vitro* and effectively impaired A128T IN HIV-1_NL4-3_ replication in infected cells, whereas in control experiments the A128T IN HIV-1_NL4-3_ exhibited marked resistance to BI-1001 (Figure S3C).

**Figure 3:**
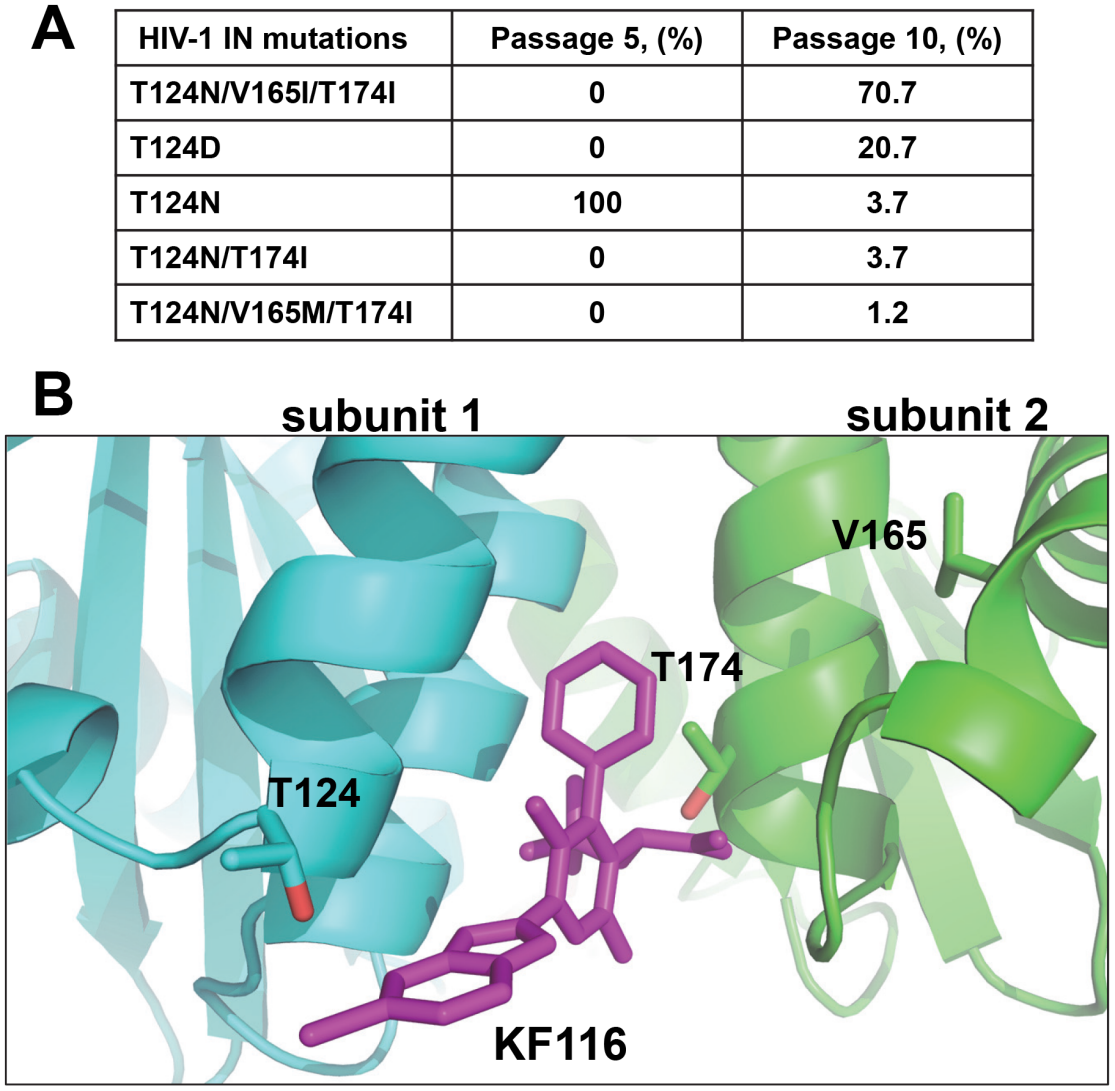
Genotype of HIV-1 variants selected in cell culture in the presence of KF116. (**A**) Mutations in the HIV-1_NL4-3_ IN gene of resistant viruses selected with KF116. Clonal sequencing of viral passage was carried out at passages 5 and 10, respectively. Eighty-two clones from each viral passage were sequenced using three sequencing primers covering the entire IN gene. Percentage of IN mutations for a given passage are indicated. Passage 5 corresponds to 50 days of selection with the KF116 concentration reaching 0.8 µM. Passage 10 corresponds to 100 days of selection with the KF116 concentration reaching 25.6 µM. (**B**) Crystal structure of KF116 bound to HIV-1 IN CCD dimer indicating the Thr-124, Val-165 and Thr-174 residues. The IN subunit 1 and 2 are colored in cyan and green, respectively. KF116 is shown in magenta.

To select HIV-1 strains resistant to KF116, HIV-1_NL4-3_ was passaged serially in MT-4 cells under increasing concentrations of the inhibitor as described [45]. Clonal sequencing of KF116-selected viruses after 5 and 10 successive passages revealed substitutions in HIV-1 IN (Figure 3A). A single T124N substitution emerged after 5 passages, with KF116 concentration reaching 0.8 µM. With further increases in KF116 concentrations, which reached 25.6 µM at passage 10, the T124N substitution within the viral pool diminished to ∼3.7% and instead the triple (T124N/V165I/T174I) substitution in HIV-1 IN emerged (Figure 3A). As expected (Figure S3) the A128T substitution, which is sufficient to confer resistance to BI-1001 [39], [40], was not observed with KF116. Figure 3B shows that all of the substitutions selected under KF116 pressure were located within or near the KF116 binding site thus paralleling the structural results (Figure 2D) in the context of infected cells.

### KF116 affects HIV-1 virion core morphology and inhibits subsequent reverse transcription in target cells

**Figure 4:**
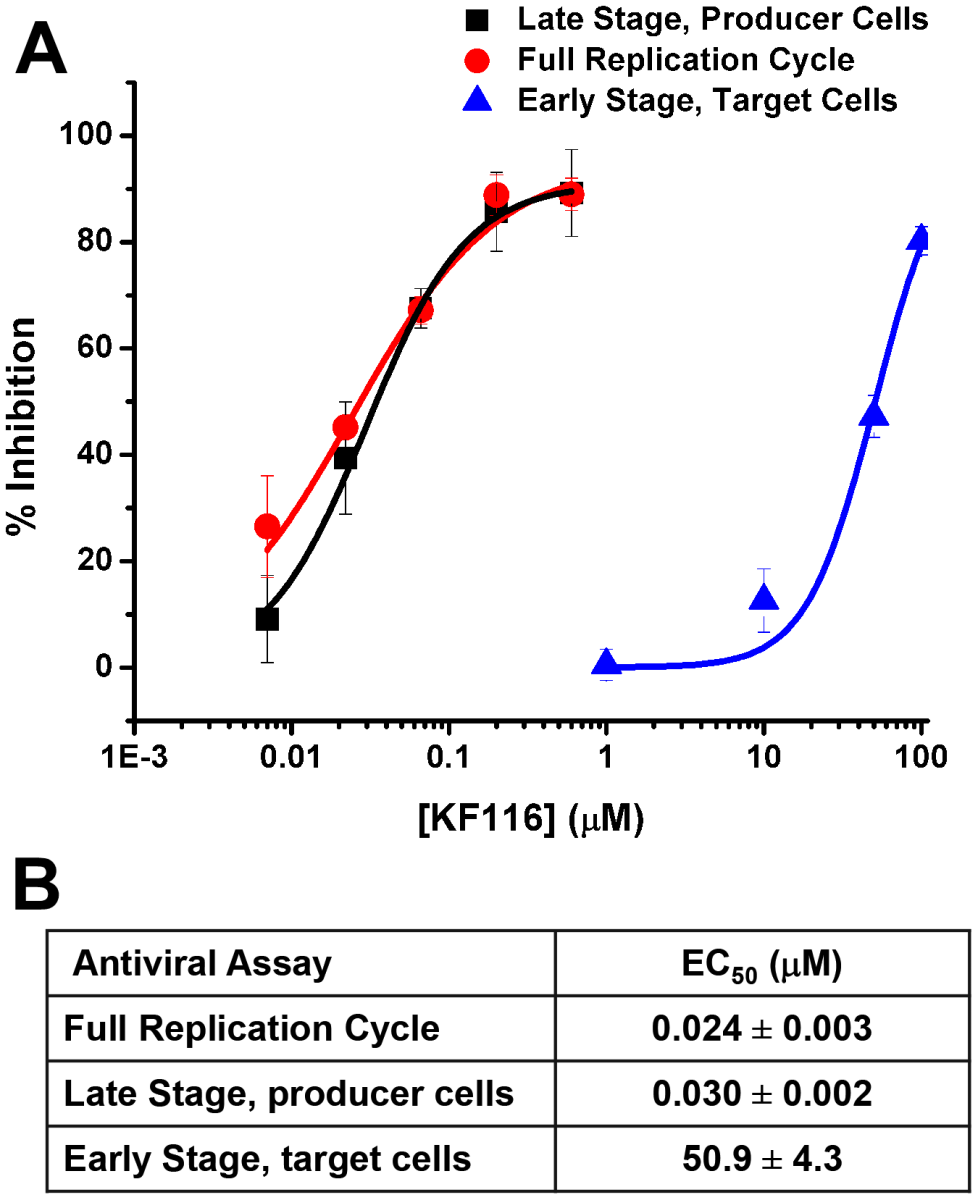
KF116 selectively impairs the late stage of HIV-1 replication. (**A**) Dose-response curves for KF116 antiviral activities during early stage, late stage or one full replication cycle. For early stage experiments, KF116 was added directly to the target cells and then these cells were infected with untreated virions. For late stage experiments, the progeny virions were prepared in the presence of KF116 and were then used to infect untreated target cells. For one full replication cycle experiments, KF116 was added to both producer and target cells. (**B**) EC_50_ values for the indicated antiviral assays. Results represent mean ± SD from three independent experiments.

To dissect the primary mechanism of KF116 inhibition, we have examined its effects on early and late stages of HIV-1 replication by adding the inhibitor to target or producer cells. When added to the producer cells KF116 inhibited HIV-1 replication with an IC_50_ of ∼0.03 µM, which closely correlated with the IC_50_ values obtained in full replication cycle (∼0.024 µM, Figure 4). In contrast, KF116 was ∼2,000-fold less effective in target cells (Figure 4). Since the secondary mechanism of action of KF116 in target cells is observed at the inhibitor concentrations that significantly exceeds a clinically relevant (submicromolar) range, our mechanistic studies have focused on the primary mechanism of action of KF116 seen in producer cells.

**Figure 5:**
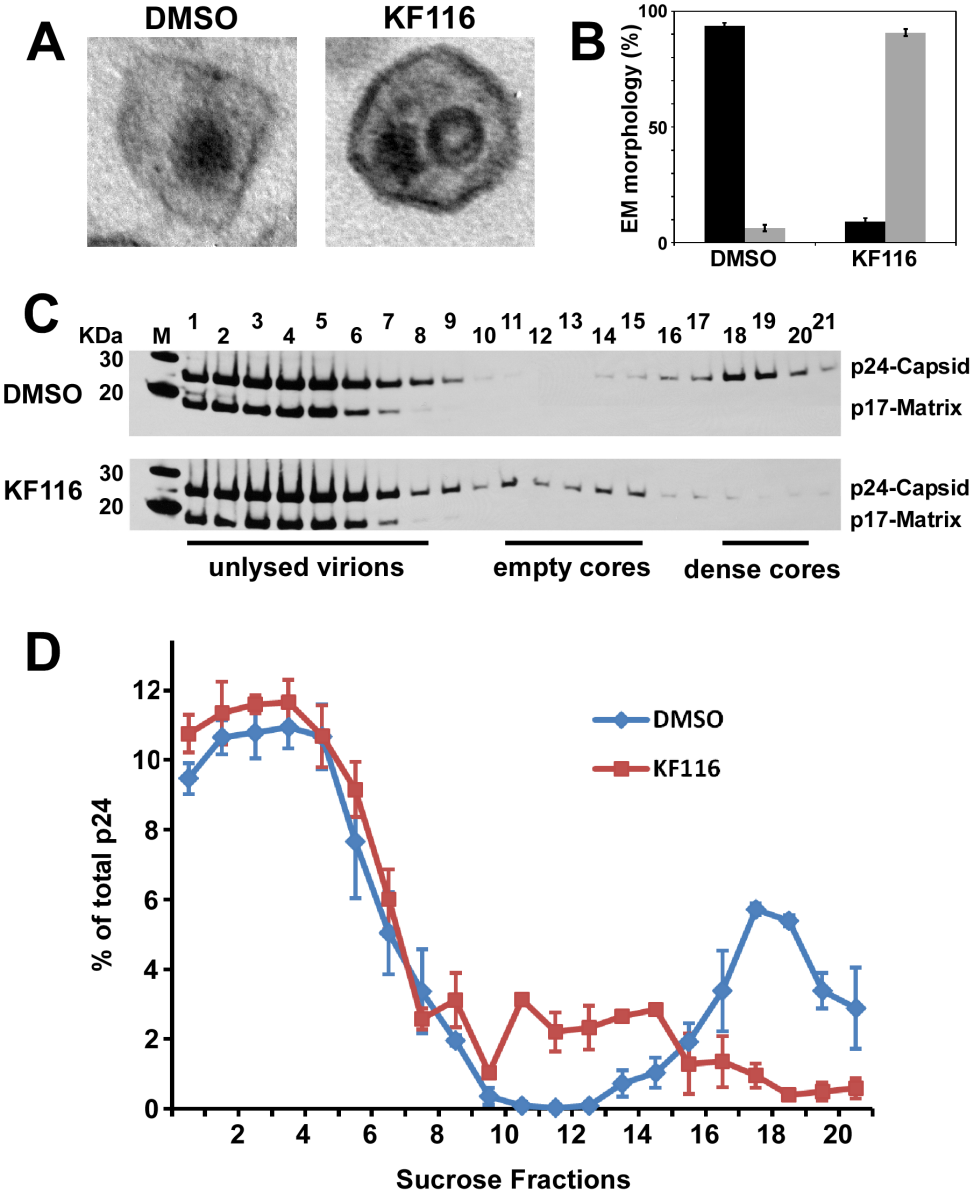
KF116 impairs formation of dense cores in HIV-1 virions. (**A**) Representative thin-section electron micrographs of HIV-1 virions produced in the presence of DMSO or 1 µM KF116. (**B**) Quantitative analysis of mature virions prepared in the presence of DMSO or 1 µM KF116. Correctly matured electron dense cores are shown in black and eccentric virions lacking electron density are shown in gray. Standard errors determined from two independent experiments are shown. Images of at least 50 mature virions were examined from each experiment. (**C**) Sucrose density gradient fractionation of detergent-lysed HIV-1 virions produced in HEK293T cells in the presence of DMSO or 1 µM KF116. Cell-free virions were harvested, detergent-lysed, and separated on 30–70% linear sucrose density gradients. Twenty-one 0.5 ml fractions were collected from the top of the gradient and subjected to SDS-PAGE and immunoblotted with HIV-1 Gag antisera to monitor the distribution of HIV-1 capsid. Positions of Gag p24 (capsid) and Gag p17 (matrix) are indicated. (**D**) Quantitation of HIV-1 capsid (p24) signal intensity from (B) as measured by ImageJ software. Graph represents the relative distribution of HIV-1 capsid (p24) in each of the sucrose density gradient fractions.

The data in Figures S4 and S5 demonstrate that KF116 treatment did not affect virus particle production, HIV-1 Gag/Gag-Pol protein processing, and viral genomic RNA packaging. Examination of virion morphology with thin-section transmission electron microscopy revealed that treatment of virus-producer cells with KF116 impaired the formation of electron-dense cores and resulted in virions with conical cores that were devoid of electron dense RNPs (referred here to as eccentric cores). Instead, the RNPs were mislocalized between the core and viral membrane (Figure 5A), similar to eccentric HIV-1 viral particles produced upon ALLINI treatments [38], [41], [42] or with select IN class II mutants [46]–[49]. Quantitative analysis of mature virions have revealed a marked increase in eccentric cores (∼95%) upon KF116 treatments compared with virions produced in the absence of the inhibitor (∼6%) (Figure 5B). Analytical sucrose density gradient fractionation of detergent-lysed virions and immunoblot analyses with HIV-1 Gag antisera have similarly revealed that the KF116 treatment resulted in reduction (>95%) of HIV-1 capsid (p24) in higher density fractions (compare fractions 18–20 in the absence and presence of KF116 in Figures 5C and 5D). These results suggest that the density of the viral cores decreased upon inhibitor treatment and are consistent with formation of an empty core due to mislocalization of the RNPs (Figure 5A).

To examine whether mislocalization of RNPs could affect the initiation of reverse transcription, the extension of tRNA^Lys3^ primer was measured using total RNA isolated from KF116 or DMSO treated virions and recombinant reverse transcriptase (RT). Figure S5A shows similar levels of extension products in the inhibitor treated and untreated control samples suggesting that KF116 did not significantly affect annealing of tRNA^Lys3^ primer to the cognate viral RNA template. Furthermore, experiments in Figure S6B have shown that KF116 had no effects on virion-associated RT activities. These findings are consistent with a previous report showing that ALLINI GS-B did not detectably affect endogenous RT activity [41].

**Figure 6:**
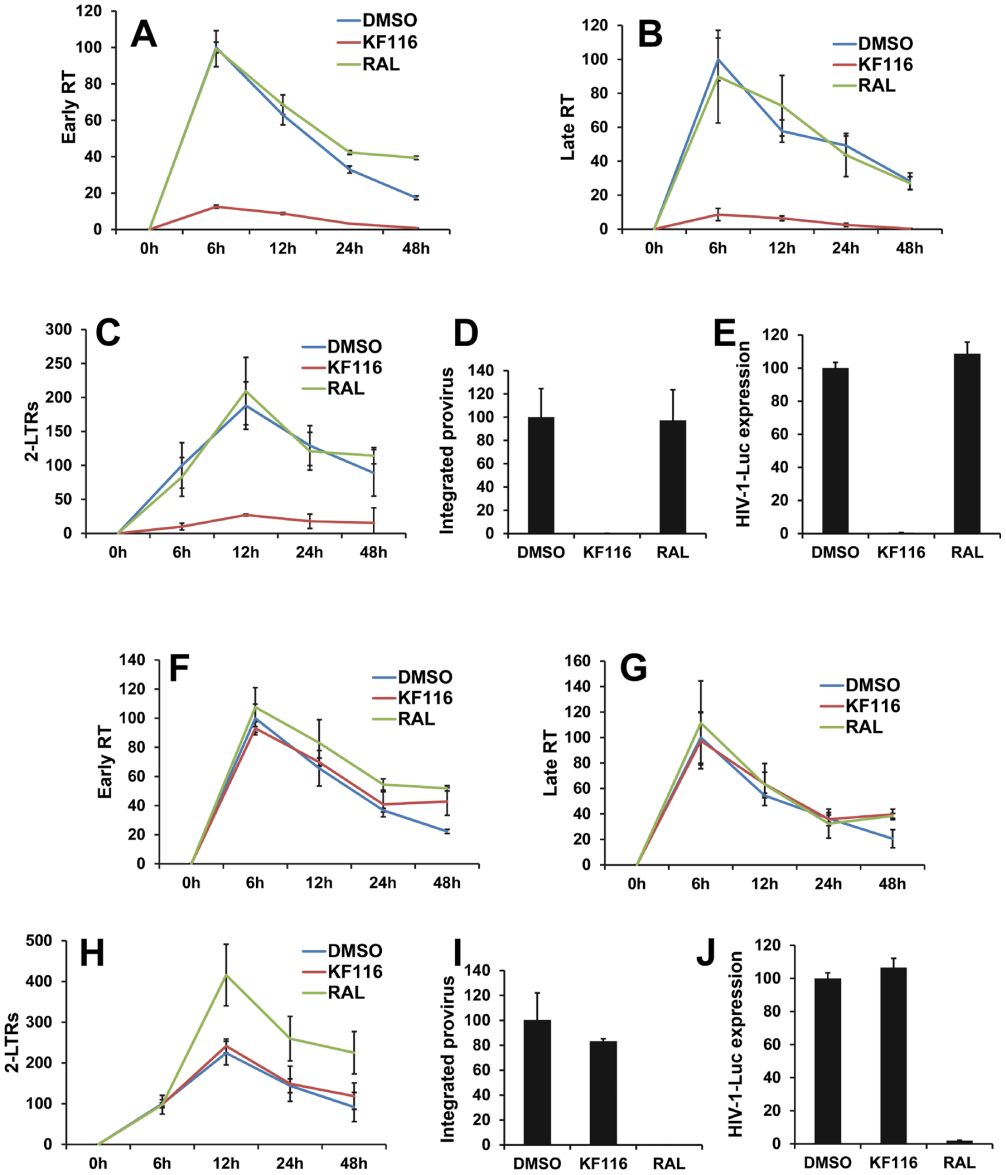
Virions produced in the presence of KF116 are defective in reverse transcription. (**A–E**) VSV-G pseudotyped HIV-1-Luc produced in the presence of DMSO, 1 µM KF116, or 1 µM RAL were used to infect HEK293T cells. Infected cells were harvested at the indicated times and subjected to quantitative PCR (qPCR) or luciferase assay. Graphs indicate the amount of PCR products relative to non-treated (DMSO) sample at 6 h post-infection for (**A**) early reverse transcription (Early RT), (**B**) late reverse transcription (Late RT) and (**C**) 2-LTR circles (2-LTRs) products. (**D**) Bar graphs indicate the integrated provirus relative to non-treated (DMSO) control at 7 days post-infection. (**E**) Aliquots of infected cells were harvested and luciferase assay was performed at 48 h post-infection. The luciferase signal obtained for the non-treated (DMSO) sample was set to 100%. All graphs represent mean ± SD (*n* = 3). (**F–J**) HEK293T cells were treated with DMSO, 1 µM KF116, or 1 µM RAL and then infected with VSV-G pseudotyped HIV-1-Luc. Infected cells were harvested at the indicated times and subjected to qPCR or luciferase assay. Graphs indicate the amount of PCR products relative to non-treated (DMSO) sample at 6 h post-infection for (**F**) early reverse transcription (Early RT), (**G**) late reverse transcription (Late RT) and (**H**) 2-LTR circles (2-LTRs) products. (**I**) Bar graphs indicate the integrated provirus relative to non-treated (DMSO) sample at 7 days post-infection. (**J**) Aliquots of infected cells were harvested and luciferase assay was performed at 48 h post-infection. The luciferase signal obtained for the non-treated (DMSO) sample was set to 100%. All graphs represent mean ± SD (*n* = 3).

We monitored how KF116 treatment of virus-producer cells affected subsequent early replication steps in target cells. For these experiments we used 1.0 µM inhibitor, which would allow us to distinguish the primary mechanism of action of KF116 (IC_50_ of ∼0.030 µM) seen in producer cells from the weaker activity (IC_50_ of ∼50 µM) detected in target cells. Figure S7 shows that comparable levels of genomic RNA were detected in target cells when infected by equivalent numbers of particles prepared in the presence or absence of KF116 suggesting that the mislocalization of RNPs upon KF116 treatment does not significantly compromise viral entry. Next, we have examined levels of early and late RT products as well as 2-LTR circles and integrated proviruses using quantitative PCR (qPCR). In control experiments, the selective IN strand transfer inhibitor RAL was tested. Producing virions in the presence of RAL yielded no inhibitory effects in the target cells (Figure 6A–E), but virions produced in the presence of KF116 exhibited sharply reduced levels of early and late RT products (Figure 6A and 6B). The markedly defective viral DNA synthesis resulted in reduced levels of 2-LTRs, integrated proviruses and subsequent viral expression (Figure 6C–E).

For control experiments KF116 or RAL were added to the target cells and the viral replication intermediates were monitored after infection with untreated virions (Figure 6F–J). As expected, RAL selectively inhibited the integration step and increased the levels of 2-LTRs (Figure 6H and 6I). In contrast, treatment with 1 µM KF116 had no effects on reverse transcription, integration, levels of 2-LTRs, or viral expression (Figure 6F–J). These findings are consistent with the results in Figure 4 indicating that KF116 is not an effective inhibitor of HIV-1 when added to target cells.

### KF116 promotes aberrant IN multimerization *in vitro* and in virus particles

Our subsequent efforts have focused on delineating the contributions of aberrant IN multimerization versus the potential inhibition of IN-LEDGF/p75 binding on the antiviral activities of KF116. Homogeneous time-resolved fluorescence (HTRF)-based IN multimerization assays were used to determine EC_50_ values of KF116 and ALLINIs for promoting IN multimerization *in vitro*. However, this technique cannot delineate multimeric forms of IN formed in the presence of inhibitors [33], [35] and some groups have proposed that ALLINIs stabilize an inactive dimer of IN [35], while others have suggested the formation of higher order IN multimers [40]. To better understand KF116 induced IN multimerization, we employed dynamic light scattering (DLS), an optical method used for determining the diffusion coefficients of particles in solution [50]–[52]. Since the diffusion coefficient depends on the particle size and shape, DLS can be used to study particle aggregation [51], [53], [54].

**Figure 7:**
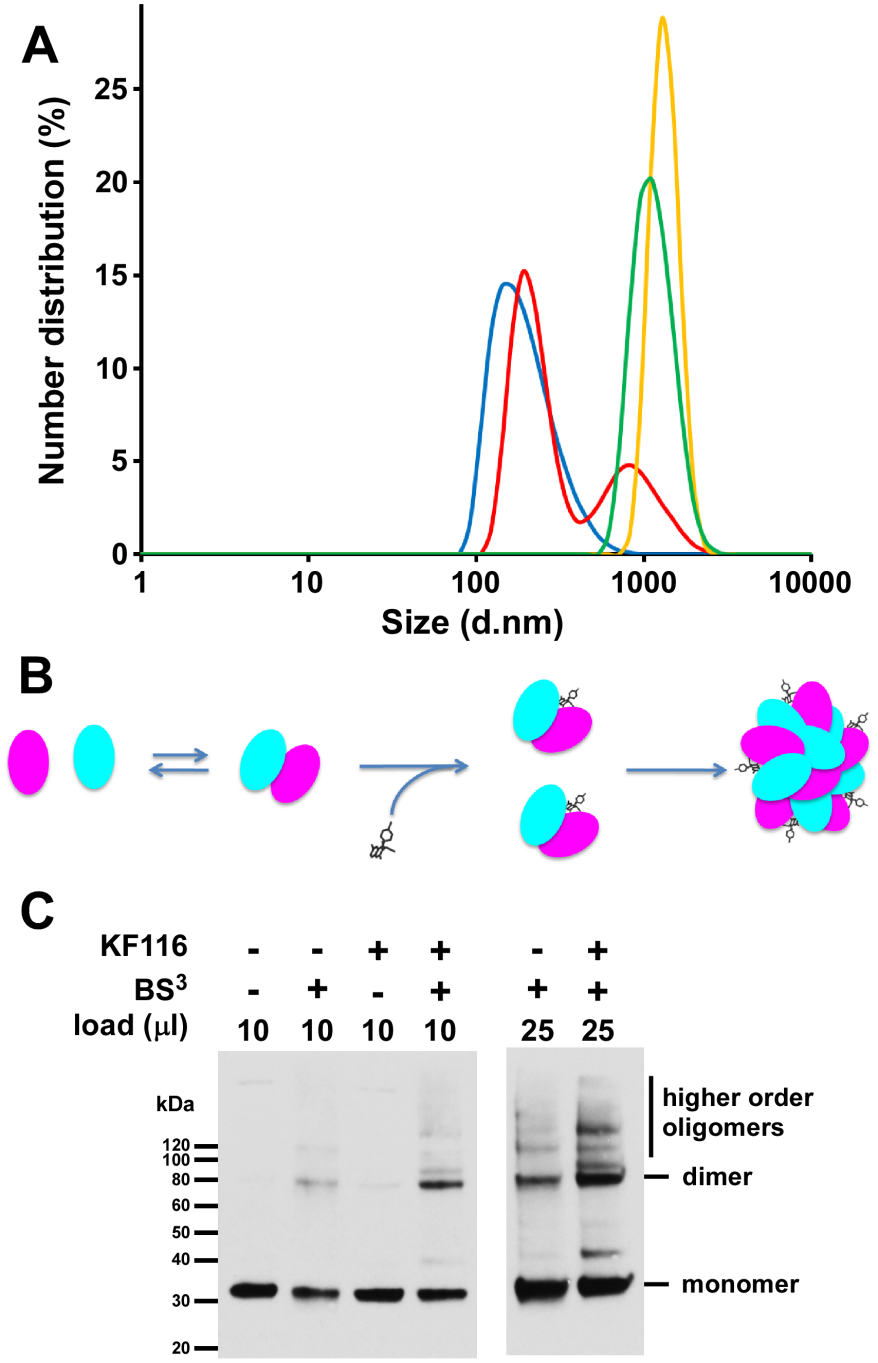
KF116 promotes IN multimerization *in vitro* and in HIV-1 particles. (**A**) DLS analysis of KF116-induced multimerization of recombinant IN. Size distribution of IN at 2 minutes (blue), 8 minutes (red), 30 minutes (green) and 120 minutes (yellow) after addition of KF116. No detectable signal was recorded in control experiments with KF116 alone or IN+DMSO incubated for up to 120 minutes. (**B**) The schematic to show the inhibitor induced equilibrium shift toward aberrant IN oligomerization. (**C**) HIV-1 virions were produced in HEK293T cells in the presence of DMSO or 1 µM KF116, cell-free virions were harvested, detergent-lysed, and treated with BS^3^ cross-linking reagent. The indicated volumes of cross-linked reaction products were resolved by SDS-PAGE and immunoblotted with HIV-1 IN antibody. The bands corresponding to IN “monomer”, “dimer”, and “higher order oligomers” are indicated.

In the absence of added inhibitor, 0.2 µM IN did not yield any signal presumably due to the relatively small size of the fully soluble protein (Figure 7A). In control experiment with only KF116, no signal was recorded indicating that the inhibitor does not self-aggregate in the aqueous solution (Figure 7A). Immediately upon addition of KF116 to IN, we observed the appearance of a peak corresponding to a particle size with 142 nm diameter (Figure 7A). Within 8 minutes two peaks corresponding to larger particle sizes with 190 nm and 955 nm diameters were detected. Particle sizes increased further after 30 min (1106 nm diameter) and 120 min (1281 nm diameter) incubation of IN with KF116. These results indicate the equilibrium shift toward higher order oligomers in a time-dependent manner. While we cannot not delineate the shapes of these complexes or determine the exact multimeric states of IN in the complex with KF116, our results can be compared with reported volumes of functional IN tetramers. For example, atomic force microscopy experiments have revealed that IN tetramers in the stable synaptic complex have volumes ∼220 nm^3^ [55], which correspond to a diameter of ∼7.5 nm if a spherical shape is assumed. Thus, our DLS results reveal that the sizes of IN oligomers formed in the presence of KF116 significantly exceed that of tetrameric forms of the protein [55], [56]. Figure 7B shows schematic interpretation of the DLS results. In the absence of the inhibitor, IN is in a dynamic equilibrium between monomers, dimers and tetramers (for clarity only monomers and dimers are shown in Figure 7B). KF116 binds at the IN CCD dimer interface and stabilizes interacting IN subunits, which in turn shifts the equilibrium toward aberrant, higher order oligomerization. Such a mechanism is also consistent with the marked increases in HTRF signal observed upon KF116 addition to IN (Figure S2).

To monitor IN multimerization in virions from KF116 treated cells we used the bifunctional BS^3^ cross-linking reagent [41]. Under the mild cross-linking conditions used in our experiments we observed monomers and dimers of IN when virions from non-treated cells were subjected to cross-linking reactions (Figure 7C). When virions from KF116 treated cells were subjected to similar cross-linking reactions, the levels of IN dimers increased and new slower migrating bands were observed which likely corresponded to higher oligomeric states of IN. Collectively, these findings (Figure 7) demonstrate that KF116 promotes aberrant IN multimerization both *in vitro* and in virus particles.

### KF116 does not effectively inhibit IN-LEDGF/p75 interactions in infected cells

To assess the effects of KF116 on IN-LEDGF/p75 interactions we used i) a LEDGF/p75 knockout (KO) HEK293T cell line to determine the KF116 IC_50_ values for target and producer cells; and ii) ligation-mediated PCRs coupled with 454-pyrosequencing experiments to examine whether KF116 affects LEDGF/p75-dependent targeting of HIV-1 integration site distribution on chromatin.

**Figure 8:**
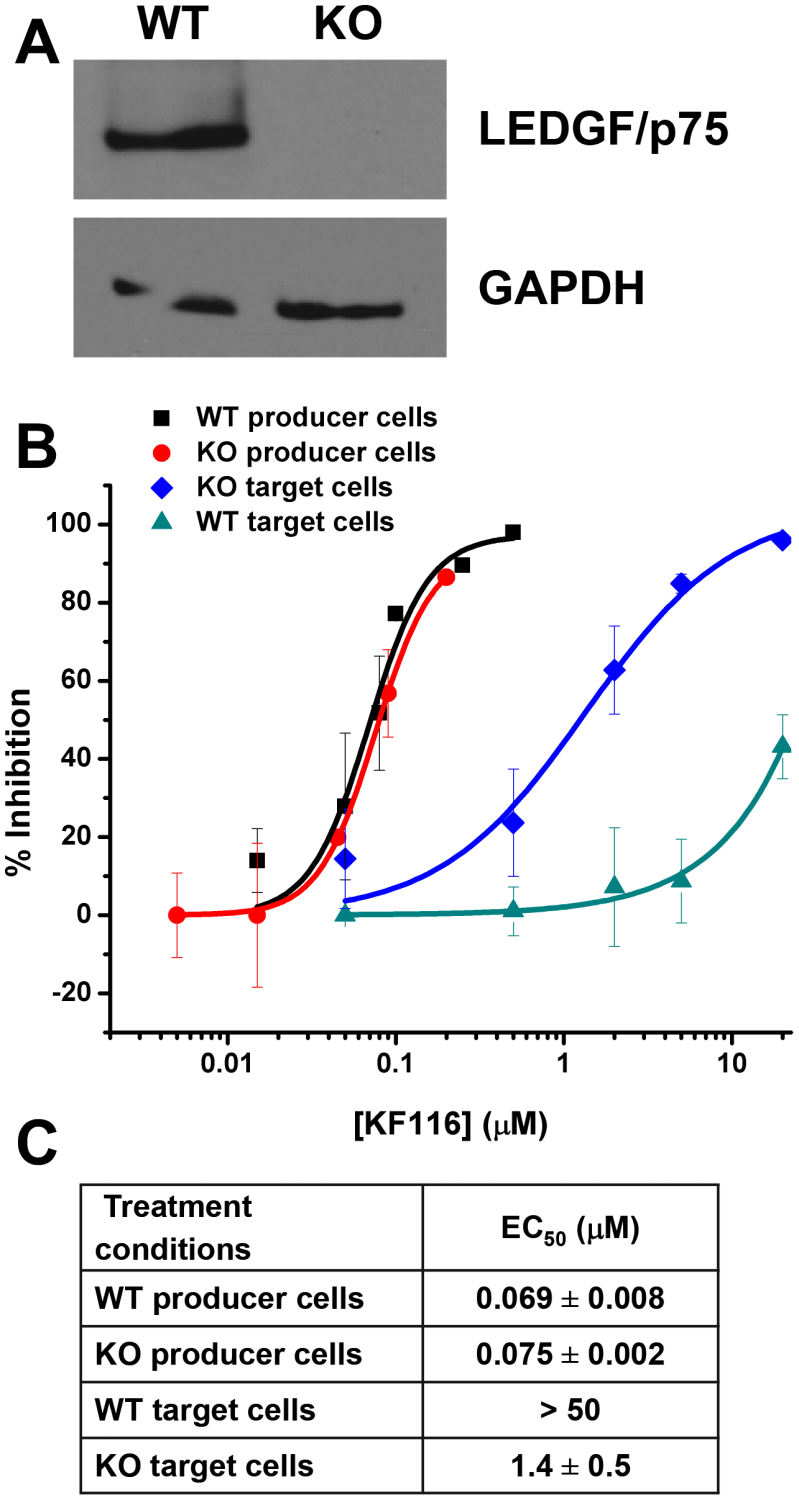
LEDGF/p75 expression does not affect KF116 potency during late stage of HIV-1 replication. (**A**) Equivalent whole cell lysates from the clonal TALEN-derived *PSIP*1 KO cell line (indicated as “KO”) and parental wild type HEK293T cell line (indicated as “WT”) were subjected to SDS-PAGE and immunoblotted for LEDGF/p75 and a GAPDH control to verify knockdown of LEDGF/p75 protein. (**B**) Dose-response curves representing the antiviral assays performed in WT or KO cell lines under the indicated conditions of drug treatment. For producer cell treatment, the VSV-G pseudotyped HIV-1-Luc progeny virions were prepared in the indicated cell line in the presence of KF116 and were then used to infect untreated HEK293T cells. For target cell treatment, KF116 was added directly to the indicated cell line and the cells were infected with untreated VSV-G pseudotyped HIV-1-Luc virions. (**C**) EC_50_ values for the indicated antiviral assays. Results represent mean ± SD from three independent experiments.

Previous studies [38], [41] have shown that siRNA- or shRNA-based LEDGF/p75 knockdown (KD) does not significantly affect IC_50_ values of quinoline-based ALLINIs when the inhibitor is added to the virus-producing HEK293T cells. In the present study we used a *PSIP1* (LEDGF/p75 gene) KO HEK293T cell line in which site-specific gene targeting with TALENs was used to engineer a gene deletion spanning exon 2 to 14 [57]. These cells thus lack a 42 kb chromosome 9 segment that contains all exons encoding the IBD and LEDGF protein domains N-terminal to it. Immunoblot analysis verified absence of LEDGF/p75 protein in the *PSIP1* KO cells (Figure 8A). We compared the IC_50_ values of KF116 in HEK293T cells containing wild type (WT) levels of LEDGF/p75 versus LEDGF/p75 KO cells by adding the inhibitor to target or producer cells (Figure 8B and 8C). In target cells KF116 exhibited significantly higher potency in LEDGF/p75 KO cells (IC_50_ of ∼1.4 µM) compared with WT cells (IC_50_ of >50 µM) suggesting that LEDGF/p75 effectively competes with KF116 for binding to IN during early steps of infection. However, the IC_50_ values of KF116 in target and producer *PSIP1* KO cells still differed significantly (Figure 8B and 8C, compare IC_50_s of ∼1.4 µM versus ∼0.075 µM for target versus producer LEDGF/p75 KO cells). When KF116 IC_50_ values were examined in producer cells, they remained unaffected by the *PSIP1* KO (IC_50_ of ∼0.069 µM for WT vs. ∼0.075 µM for KO cells) indicating that the presence of LEDGF/p75 during late stage of HIV-1 replication does not influence KF116 potency (Figure 8).

**Figure 9:**
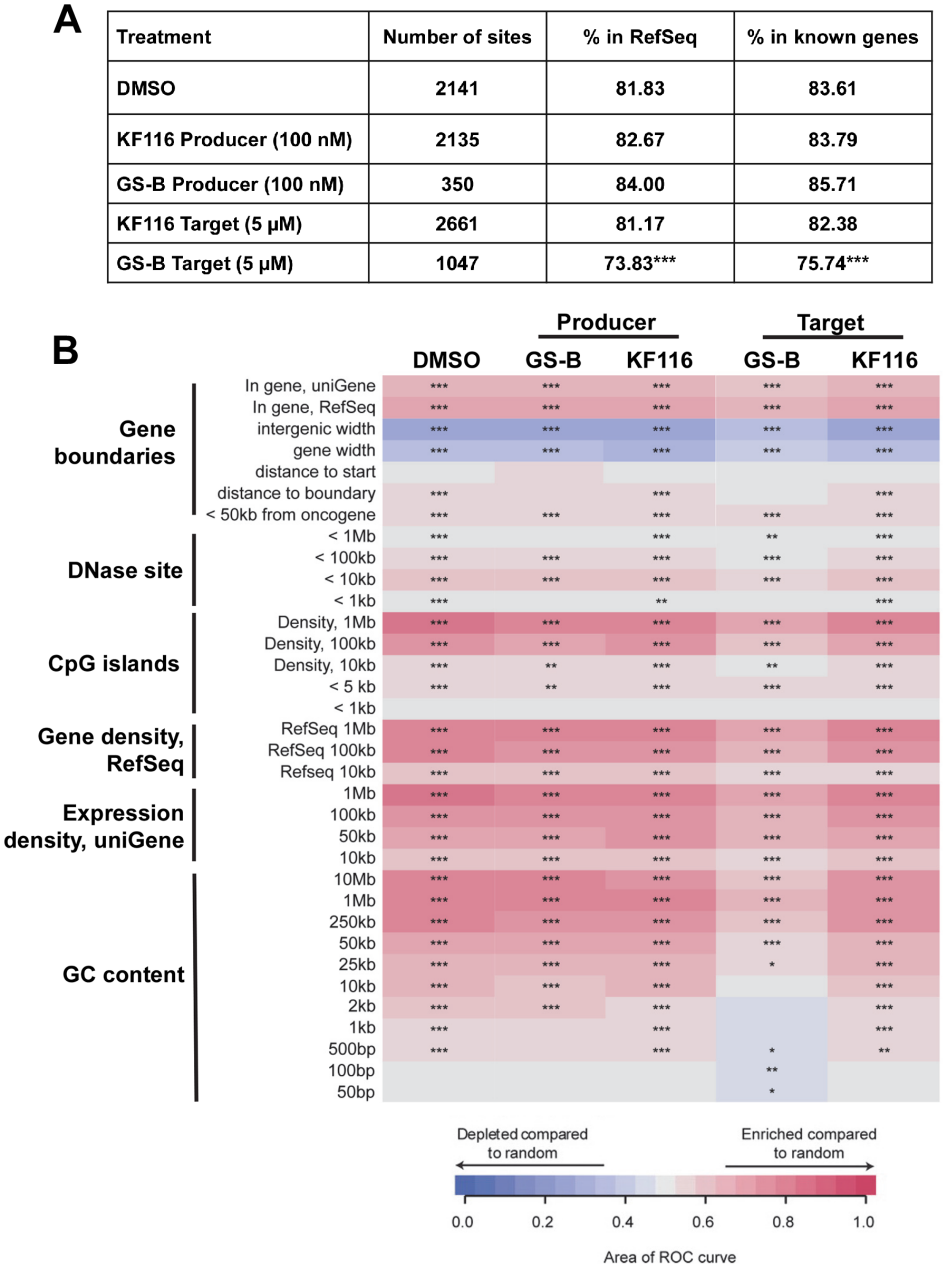
Effects of MINI KF116 and ALLINI GS-B on LEDGF/p75-dependent targeting of HIV-1 integration site distribution on chromatin. (**A**) HIV-1 integration frequencies in RefSeq and known genes, comparing effects of KF116 and GS-B on LEDGF/p75-dependent targeting of HIV-1 integration. All the samples differed significantly from their respective matched random controls (using Fisher's exact test, *P<0.001*). Significant deviation from the control non-treated (DMSO) sample was calculated using Fisher's exact test and is denoted by asterisks (*** *P<0.001*). (**B**) Heatmap summarizing HIV-1 integration frequencies relative to the genomic features. The columns depict HIV-1 integration site data sets for the indicated drug treatments. For experiments involving the drug treatment of virus-producer cells (indicated “Producer”), the progeny virions were prepared in the presence of 0.1 µM KF116 or 0.1 µM GS-B and were then used to infect untreated target cells. For experiments involving the drug treatment of target cells (indicated “Target”), 5 µM KF116 or 5 µM GS-B inhibitor was added directly to the target cells and the cells were infected with untreated virions. The rows depict the analyzed genomic features and the base pair values shown in the rows indicate the size of a given genomic interval used for the analysis. The relationship between HIV-1 integration site frequencies relative to matched random controls for each of the genomic feature was quantified using the receiver operator characteristic (ROC) curve area method. The color key shows enrichment (indicated in red) or depletion (indicated in blue) of a given genomic feature near integration sites. *P* values were calculated for the individual integration site data sets compared to the matched random controls, ***P<0.001; **P<0.01; *P<0.05.

LEDGF/p75 is the primary cellular factor that directs HIV-1 integration into active genes [58], [59]. Therefore, potential inhibitors of IN-LEDGF/p75 interactions would be expected to result in reduced HIV-1 integration frequencies into active genes. We monitored HIV-1 integration site distributions in cells treated with KF116 or GS-B (KF116 EC_50_ of ∼0.024 µM vs. GS-B EC_50_ of ∼0.026 µM for full replication cycle). The concentrations of KF116 and GS-B were optimized so that sufficient integration sites would be generated for statistical analyses. For the virus-producer cell treatments, two concentrations of KF116 or GS-B were tested, 0.1 µM and 0.2 µM. The progeny virions produced in the presence of 0.2 µM inhibitor were not sufficiently infectious to enable subsequent 454-pyrosequencing experiments. In contrast, treatments with 0.1 µM of KF116 or GS-B yielded ∼77% reduction in target cell HIV-1 expression when compared to the untreated virions (expression here serves as a measure of provirus formation) and allowed us to monitor genome wide integration site distribution (Figure 9). As expected [58], [59] with DMSO control experiments HIV-1 integration was significantly favored into active genes (RefSeq and known genes, Figure 9). When compared to the DMSO control, no statistically significant differences were observed in HIV-1 integration into active genes when KF116 or GS-B was added to producer cells.

Because of the reduced potencies of these inhibitors in target cells, 5 µM and 10 µM GS-B were used, which resulted in ∼71% and ∼88% inhibition of HIV-1 expression, respectively. Under these conditions statistically significant reduction of HIV-1 integration frequencies into RefSeq and known genes were observed (Figure 9 and Figure S8, *P<0.001*). Therefore, we have also examined matching concentrations (5 µM and 10 µM) of KF116 in target cells. At these concentrations, KF116 did not significantly inhibit HIV-1 expression (Figure 4) or result in any significant changes in HIV-1 integration frequencies into RefSeq and known genes (Figure 9 and Figure S8). Taken together, our results show that KF116 primarily inhibited HIV-1 replication with low nanomolar IC_50_ values by inducing aberrant IN multimerization.

## Discussion

### Rational design of MINIs

The quinoline-based ALLINIs are promising leads for the development of clinically useful HIV-1 IN inhibitors. However, the multifunctional nature of quinoline-based ALLINIs, which inhibit IN-LEDGF/p75 binding and promote aberrant IN multimerization with similar potencies *in vitro*, has caused controversy about their antiviral mechanism of action. For example, it has been proposed that these compounds act through selectively inhibiting IN-LEDGF/p75 binding in infected cells [32], whereas others studies argued that ALLINIs impair HIV-1 replication primarily through inducing aberrant IN multimerization [33]–[35], [38]. Furthermore, the A128T mutation in IN has been shown to confer marked resistance to the majority of ALLINIs. Here we report a new class of pyridine-based MINIs by rationally modifying quinoline-based ALLINIs. We also note that independently from our present studies, Boehringer Ingelheim reported structurally related compounds as inhibitors of HIV-1 replication in a patent application [60]. However, the patent did not describe the mechanisms of action or structural basis for their interactions with HIV-1 IN.

Our structural and mechanistic studies have revealed the following differences between MINIs and ALLINIs. *In vitro* assays have shown that unlike ALLINIs, which exhibit similar IC_50_ values for inhibiting IN-LEDGF/p75 binding and promoting aberrant IN multimerization, pyridine-based KF115 and KF116 modulate IN multimerization but are not effective inhibitors of IN-LEDGF/p75 binding (Table 1). Our structural studies have provided a plausible explanation for these observations. Unlike the rigid, planar quinoline ring in ALLINIs, the pyridine-based design contains a rotatable single bond which allows the benzene ring of KF115 or the benzimidazole ring of KF116 to engage subunit 1 through rotation out of the plane of the pyridine core. These additional interactions allow KF115 and KF116 to bridge the two IN subunits more effectively than their ALLINI counterparts. Consequently, the pyridine-based compounds exhibit significantly higher selectivity for promoting aberrant IN multimerization.

In the absence of an added ligand, interactions between IN subunits are highly dynamic [16]. Viral DNA ends mediate ordered IN multimerization by allowing individual subunits to assemble into a functional tetramer in the context of the SSC [27]. LEDGF/p75 bridges between both individual subunits and two IN dimers to stabilize a tetrameric form of IN [16]. In contrast, small pyridine-based compounds promote subunit-subunit interactions and shift the equilibrium to higher order oligomers. Our results (Figure 7) show that upon addition of KF116 the oligomeric state of IN increased in a time-dependent manner leading to aberrant IN multimerization.

The planar quinoline core found in ALLINIs extends toward the Ala-128 residue and allows the evolution of HIV-1 IN A128T escape mutation. In contrast, the benzimidazole ring in KF116 is oriented perpendicular to the core pyridine ring (Figure 2), which enables this compound to avoid the steric effects of the A128T substitution seen with archetypal ALLINI BI-1001 [40]. While a single A128T mutation was sufficient to confer marked resistance to a number of ALLINIs, selection of HIV-1 variants under genetic pressure of KF116 revealed the triple T124N/V165I/T174I mutant as the predominant variant. Thr-124 and Thr-174 lie in the KF116 binding site, while Val-165 is distant from the inhibitor binding site and is involved in inter-protein interactions. Therefore, it is possible that T124N and T174I substitutions directly affect KF116 binding, while the V165I substitution could be a compensatory mutation to restore IN function and/or stability that may be compromised by the T124N and T174I substitutions. This hypothesis would suggest an increased genetic barrier imposed by KF116 in comparison to ALLINIs.

### KF116 is not effective during early stages of HIV-1 replication

Comparative analyses of antiviral mechanisms of action of MINIs and ALLINIs are of interest for delineating the effects of aberrant IN multimerization versus inhibiting IN-LEDGF/p75 interactions in infected cells. The main differences between KF116 and GS-B were seen in target cells, where the IC_50_ values of GS-B (∼0.7 µM) [41] versus KF116 (>50 µM) differed markedly. The reduced activities of ALLINIs in target cells compared with activity against the full replication cycle can be explained by endogenous LEDGF/p75 competing with these inhibitors during early infection steps. Indeed, ALLINI IC_50_ values in LEDGF/p75 KD target cells correlated closely to their EC_50_ values in full replication assay [38]. Our results in Figure 8 demonstrate that LEDGF/p75 also competes with KF116, but even upon removal of LEDGF/p75, the KF116 EC_50_ in LEDGF/p75 KO target cells was still ∼20-fold higher than the IC_50_ in the full replication assay, implicating LEDGF/p75-independent inhibition of late steps as the primary mechanism of inhibition.

Previous reports have shown that addition of GS-B to target cells does not affect reverse transcription but blocks HIV-1 integration [35], [41]. By monitoring the frequencies of deletions and insertions at the 2-LTR junctions Tsiang *et al.* concluded that GS-B affected HIV-1 replication at a step preceding 3′-processing by inducing IN multimerization, which in turn compromised the formation of the functional IN-viral DNA complex [35]. *In vitro* experiments have demonstrated that ALLINIs can potently inhibit both the formation of the SSC and the LEDGF/p75 binding to the SSC [33]. Therefore, we wanted to explore whether GS-B could also affect IN-LEDGF/p75 binding in infected cells. The treatment of target cells with 5 µM or 10 µM GS-B resulted in statistically significant reduction of HIV-1 integration in active genes. Since LEDGF/p75 is the primary cellular factor responsible for targeting HIV-1 integration to actively transcribed units [58], [59], our findings indicate that the treatment of target cells with GS-B likely inhibits IN-LEDGF/p75 binding in the infected cells. These results extend previous observations invoking allosteric modulation of the IN structure by GS-B [35]. Collectively, our findings together with the published results [33]–[35] argue for a multimodal mechanism of action of ALLINIs in the infected cells and are consistent with *in vitro* results. In contrast to GS-B treatment, the treatment of target cells with 5 µM or 10 µM KF116 had no measureable effect on the distribution of integration sites in the host chromatin indicating that KF116 is not an effective inhibitor of IN-LEDGF/p75 binding in infected cells. Taken together, the differential activities of KF116 and GS-B in target cells can be explained by the relative potency of GS-B compared to KF116 in impairing the IN-LEDGF/p75 interaction.

A recent report [61] has shown that the treatment of infected cells with suboptimal concentrations of RAL leads to aberrant HIV-1 integration, which results in significant rearrangements in the host genome. These findings have raised the concern that patients exposed to suboptimal doses of RAL could encounter significant unintended consequences. Our findings that KF116 at therapeutically relevant (submicromolar) concentrations does not detectably affect HIV-1 integration while effectively inhibiting morphogenesis of infectious virions during late stages of HIV-1 replication provide further impetus for developing MINIs for potential clinical applications.

### KF116 primarily inhibits the late stage of HIV-1 replication by promoting IN multimerization

The treatment of virus-producer cells with KF116 and GS-B resulted in comparable EC_50_ values of 0.030 µM and 0.039 µM, respectively, which correlate closely with their respective EC_50_ values in the full replication cycle. Previous studies have suggested that ALLINIs modulate IN multimerization during viral particle maturation [38], [41]. However, IN-LEDGF/p75 binding in the late stage of HIV-1 replication could not be discounted.

KF116 promotes aberrant IN multimerization but is not an effective inhibitor of IN-LEDGF/p75 binding, allowing its use as a probe. The KF116 EC_50_ value in the virus-producer cells closely correlated with the *in vitro* EC_50_ values for aberrant IN multimerization but not for its IC_50_ value for IN-LEDGF/p75 binding. Furthermore, viral particles prepared in the presence of KF116 displayed aberrant IN multimerization and mislocalization of viral conical cores lacking RNPs.

The genome-wide HIV-1 integration site analysis and the experiments with *PSIP1* gene KO cells allowed us to explore the role of potential IN-LEDGF/p75 interactions as a target for KF116 and GS-B during the late stage of HIV-1 replication. The treatment of virus-producer cells with 0.1 µM GS-B or 0.1 µM KF116 similarly inhibited HIV-1 expression by ∼77%. Yet, the distribution of remaining integration sites did not show any statistically significant changes from untreated cells. Moreover, complete loss of LEDGF/p75 protein in the virus-producer cells did not alter the IC_50_ values of either GS-B or KF116 during the late stage of HIV-1 replication. Taken together, we conclude that both KF116 and GS-B affect viral particle maturation through promoting aberrant IN multimerization rather than inhibiting IN-LEDGF/p75 binding.

In summary, we have developed a new class of pyridine-based MINIs which allowed us to examine the role of IN multimerization independently from IN-LEDGF/p75 binding in infected cells. Our results highlight small molecule induced aberrant IN multimerization as a plausible antiviral mechanism. Furthermore, the rational approach of designing KF116 provides a path for further development of improved MINIs for potential clinical use with increased potency and higher genetic barrier for HIV-1 resistance.

## Materials and Methods

### Antiviral compounds

Syntheses of KF115 and KF116 are described in Supporting Information (Text S1 and Figure S1). BI-1001 was synthesized as described previously [33]. GS-B was a kind gift from Gilead Sciences Inc. (California, USA). Raltegravir (RAL, Cat. No. 11680), Nevirapine (NVP, Cat. No. 4666) and Saquinavir (SQV, Cat. No. 4658) were obtained from National Institutes of Health AIDS Research and Reference Reagent Program.

### Crystallization and X-ray structure determination

The HIV-1 IN CCD (residues 50–212 containing the F185K substitution) and its A128T mutant were expressed and purified as described [62]. The CCD protein was concentrated to 8 mg/ml and crystallized at 4°C using the hanging drop vapor diffusion method. The crystallization buffer contained 10% PEG 8K, 0.1 M sodium cacodylate, pH 6.5, 0.1 M ammonium sulfate, and 5 mM DTT. Cubic-shaped crystals reached 0.1–0.2 mm within 4 weeks. The A128T CCD protein was concentrated to 8.5 mg/ml and crystallized at room temperature (20°C) using the hanging drop vapor diffusion method. The crystallization buffer contained 0.1 M sodium cacodylate, pH 6.5, 1.4 M sodium acetate and 5 mM DTT. The crystals reached 0.2–0.4 mm within 1 week. The soaking buffer containing 10 mM KF115 or KF116 was prepared by dissolving the compounds in crystallization buffer supplemented with 10% dimethyl sulfoxide (DMSO). The protein crystals were soaked in the buffer for 8 hours before flash-freezing them in liquid N_2_. Diffraction data were collected at 100 F on a Rigaku Raxis 4++ image plate detector at the Ohio State University Crystallography Facility. The intensity data integration and reduction were performed with HKL2000 program. Molecular replacement program Phaser [63] in the CCP4 package [64] was used to solve the structures. The crystal structure of the catalytic core domain of HIV-1 IN [62] (PDB # 1ITG) was used as a starting model. Coot program [65] was used for the subsequent refinement and building of the structure. Refmac5 [66] of the CCP4 package was used for the restraint refinement. TLS [67] and restraint refinement were applied for the last step of the refinement. The crystals belonged to a space group P3121 with cell dimensions a = b = 72 Å and c = 65 Å, with one 18-kDa monomer in the asymmetric unit. The structures were refined to 2.05 Å for KF115 bound to the CCD, 2.20 Å for KF116 bound to the CCD, and 2.37 Å for KF116 bound to the A128T CCD. The data collection and refinement statistics are listed in Table S1. Coordinates have been deposited in the Protein Data Bank with accession numbers 4O0J, 4O55 and 4O5B.

### *In vitro* protein-protein interaction assays

HTRF-based IN multimerization and IN-LEDGF/p75 binding assays were carried out as described previously [33]. The HTRF signal was recorded using a Perkin Elmer Multimode EnSpire plate reader.

### DLS assay

For DLS experiments 1 µM KF116 or DMSO was added to 200 nM IN in 50 mM HEPES, pH 7.4 buffer containing 2 mM DTT, 2 mM MgCl_2_ and 1 M NaCl. DLS signals were recorded at room temperature using a Malvern Nano series zetasizer instrument.

### Cells, viruses, transfections, infections

HEK293T and HeLa TZM-bl cells were cultured in Dulbecco's modified eagle medium (Invitrogen), 10% FBS (Invitrogen) and 1% antibiotic (Gibco) at 37°C and 5% CO_2_. MT-4 cells were cultured in RPMI 1640 (Invitrogen), 10% FBS (Invitrogen) and 1% antibiotic (Gibco) at 37°C and 5% CO_2_. The design and construction of HEK293T cell line, in which all alleles of the *PSIP1* (LEDGF) gene were deleted using site-specific gene targeting with transcription activator-like effector nucleases (TALENs), has been reported elsewhere [57]. The TALEN-derived *PSIP1* knockout (KO) cells lack 42 kb of *PSIP1*, with the deletion extending from exon 2 to exon 14, and thus lack all exons that encode the IBD and any protein domains N-terminal to it. They were cultured in Dulbecco's modified eagle medium (Invitrogen), 10% FBS (Invitrogen) and 1% antibiotic (Gibco) at 37°C and 5% CO_2_.

All viral stocks were generated by transfecting HEK293T cells with plasmid DNA and X-tremeGENE HP (Roche) transfection reagent at 1∶3 ratio following manufacturer's protocol. Twenty-four hour post-transfection, the culture supernatant was replaced with fresh complete medium after washing once with complete medium. Forty-eight hour post-transfection, the virus containing supernatant was collected, filtered through 0.45 µm filter and, if needed, concentrated by ultracentrifugation. Replication competent HIV-1 was generated using pNL4-3 [68]. Luciferase reporter HIV-1 (HIV-1-Luc) pseudotyped with VSV-G, was generated using pNL4-3.Luc.Env^−^ [69] and pMD.G (VSV-G envelope plasmid) [70]. HIV-1 subtype C was generated using the infectious molecular clone pMJ4 [44].

### Antiviral and cytotoxicity assays

For early stage experiments, the indicated concentrations of the test inhibitor or diluent control (DMSO) were added directly to the target cells and the cells were infected with untreated virions. Briefly, HeLa TZM-bl cells (2×10^5^ cells/well of a 6-well plate in 2 ml of complete medium) were pre-incubated with the indicated concentrations of the test inhibitor or diluent control (DMSO) for 2 h. The cells were then infected with HIV-1 virions equivalent to 4 ng of HIV-1 p24 as determined by HIV-1 Gag p24 ELISA (ZeptoMetrix) following manufacturer's protocol. Two hours post-infection the culture supernatant was removed, washed once with complete medium, and then fresh complete medium was added with the inhibitor concentration maintained. The cells were cultured for 48 h and the cell extracts were prepared using 1× reporter lysis buffer (Promega). Luciferase activity was determined using a commercially available kit (Promega).

For late stage experiments, the progeny virions were prepared in the presence of the indicated concentrations of the test inhibitor or diluent control (DMSO) and were then used to infect untreated target cells. The antiviral assays for late stage experiments were performed using the procedure previously described [40].

For full replication cycle experiments, progeny virions were prepared in the presence of the test inhibitor or diluent control (DMSO) at given concentrations. Target cells were pre-incubated for 2 h with matching inhibitor concentrations. Target cells were infected and luciferase activity was determined 48 h post infection as described above.

The cytotoxcity assays were performed as described previously [45]. The fitted dose-response curves were generated to calculate EC_50_ or CC_50_ using Origin software (OriginLab, Inc.).

### qPCR analysis

Levels of HIV-1 early reverse transcription products, late reverse transcription products (LRT), 2-LTR circles and integrated proviruses (Alu-PCR reactions) were quantified using primers, probes, and qPCR conditions described previously [71], [72]. Briefly, HEK293T cells (2×10^5^ cells/well of a 6-well plate in 2 ml of complete medium) were infected with VSV-G pseudotyped HIV-1 virions equivalent to 2 µg of HIV-1 p24 as determined by HIV-1 Gag p24 ELISA (ZeptoMetrix) following manufacturer's protocol. All viral stocks were treated with 60 U/ml DNaseI (Ambion) prior to infections to avoid plasmid DNA contamination. Infected cells were harvested at the indicated times post-infection and genomic DNA was isolated using DNeasy Blood & Tissue kit (Qiagen). For Alu-PCR reactions in HEK293T cells to quantify integrated provirus, genomic DNA was harvested from cells 7 days post-infection to eliminate unintegrated viral DNA and was used as the template for initial amplification. TaqMan-based qPCR reactions were prepared using iQ Supermix (Biorad) using primer-probe sets as described previously [71], [72]. All reactions were normalized to GAPDH using SYBR-green (iQ SYBR Green Supermix; Biorad) based qPCR and primers as described previously [71]. Relative quantification analysis was performed using the 2^−ΔΔCT^ method [73]. qPCR was performed using the CFX96 real-time system.

### Immunoblotting

Standard Western blotting procedures were used with the following antibodies: HIV-1 Gag (a gift from Kathleen Boris-Lawrie), HIV-1 RT (NIH Cat. No. 7373), HIV-1 IN (a gift from Paul Bieniasz & Michael Malim), GAPDH (Serotech AHP 1628T), and LEDGF/p75 (BD Biosciences 611714).

### Transmission electron microscopy

HIV-1 virion morphology was analyzed using thin-section transmission electron microscopy (TEM). HEK293T cells (2×10^6^ cells/100 mm culture dish in 10 ml of complete medium) were transfected with HIV-1 provirus (pNL4-3) and cultured for 24 h to ensure expression of the provirus as described above. Next, the cells were treated with the indicated inhibitor or diluent control (DMSO) for 1 h. The culture supernatant was replaced with fresh complete medium containing either the inhibitor or DMSO, and cells were then cultured for another 24 h to allow for the production of HIV-1 particles in the presence of the inhibitor or diluent control. Cells were harvested, washed twice with 1× PBS, and the cell pellets were fixed in 2.5% glutaraldehyde (in 0.1 M phosphate buffer pH 7.4 containing 0.1 M sucrose), post-fixed in 1% osmium tetroxide in the same buffer, en bloc stained in 2% uranyl acetate in 10% ethanol, dehydrated in graded ethanols, infiltrated, and embedded in Epon resin. TEM sample preparation, negative staining and imaging were performed at Campus Microscopy & Imaging Facility (Ohio State University). Images were taken with FEI Tecnai G2 Spirit transmission electron microscope.

### HIV-1 viral core analyses using sucrose density gradient fractionation

Sucrose density gradient fractionation was carried out as described previously [74] with some modifications. Briefly, HIV-1 virions were produced in the presence of the inhibitor or diluent control (DMSO) and cell-free virions were concentrated over a 25% sucrose cushion by ultra-centrifugation at 28,000 rpm for 2 h at 4°C in SW41 rotor (Beckman). Pelleted virions were resuspended in 300 µl STE buffer (10 mM Tris pH 7.5, 100 mM NaCl, 1 mM EDTA), lysed with 0.5% Triton X100 for 2 minutes at room temperature. Lysed virions were loaded on 30–70% linear sucrose density gradients in STE buffer, and ultra-centrifuged at 28,500 rpm for 16 h at 4°C in SW41 rotor (Beckman). Twenty-one 0.5-ml fractions were collected from the top of the gradient and subjected to SDS-PAGE and immunoblotted with HIV-1 Gag antisera to monitor the distribution of HIV-1 capsid.

### Resistance selection

Resistance selection was carried out as previously described [45]. Briefly, selection of resistance to HIV-1 was carried out in MT-4 cells with the inhibitor added at a final concentration corresponding to its antiviral EC_50_. Infections were initiated with HIV-1_NL4-3_ at a MOI ∼0.01. Cultures were split one-third every 3 to 4 days depending on the proliferation status of the cells. The progression of infection was monitored by the appearance of HIV-1-induced cytopathic effect (CPE, syncytia formation). When peak CPE was reached, the cell-free culture supernatant was used to infect fresh MT-4 cells in the presence of an equal or 2-fold higher inhibitor concentration. Successive viral passages were generated by repeating this procedure.

### PCR amplification, cloning and sequencing of viral DNA

Clonal sequencing of viral passage was carried out at passage 5 and 10, respectively, as described previously [45]. Total DNA isolated was from MT-4 cells infected with virus from each passage using DNeasy Blood & Tissue kit (Qiagen) and was used for the PCR amplification of a 2765 base pair viral DNA fragment spanning nucleotide 372 of the RT gene to nucleotide 127 of the Vpr gene. The PCR reaction was performed using the Platinum High Fidelity PCR SuperMix (Invitrogen) following manufacturer protocol. The PCR product was gel-purified and cloned into the pCR-XLTopo (Invitrogen). Next, 96 transformed bacterial colonies (12 from DMSO control and 84 from KF116) were cultured in a SeqPrep HP 96 Plate (EdgeBio). Plasmid DNA isolation and sequencing were carried out at Ohio State University's Plant-Microbe Genomics Facility. The entire IN gene was sequenced using the following three primers: INseq1 5′-GTCTACCTGGCATGGGTACC-3′, INseq2 5′-GTTATCTTGGTAGCAGTTCATG -3′, and INseq3 5′-CTAGTGGGATGTGTACTTCTG-3′. Sequencing reads were analyzed using BioEdit Sequence Analyzer Editor. Resistance selection under diluent control (DMSO) was used to identify naturally occurring polymorphisms. The inhibitor-specific mutations were documented based on their order of appearance and rate of emergence, as well as patterns/copurifying mutations.

### Isolation of integration sites using 454-pyrosequencing

Isolation of HIV-1 integration sites was performed using ligation-mediated PCR as previously described [75], [76] with some changes. Briefly, HEK293T cells (2×10^5^ cells/well of a 6-well plate in 2 ml of complete medium) for the indicated treatment were infected with VSV-G pseudotyped HIV-1 virions equivalent to 2 µg p24 based on HIV-1 p24 ELISA. Cells were harvested 10 days post-infection, and the genomic DNA was purified using DNeasy Blood & Tissue kit (Qiagen). Genomic DNA was fragmented with dsDNA Fragmentase (NEB) and then linkers were ligated. The provirus-host DNA junctions were amplified by nested PCR using bar-coded primers. The PCR products were gel-purified and sequenced on the 454 GS-Junior (Roche). Six independent infections were performed for each sample and samples were separately bar coded with the second pair of PCR primers to enable pooling of the PCR products for sequencing.

### Bioinformatics & statistical analyses of integration site distribution

The analysis and statistical methods for HIV-1 integration site distribution have been previously described [77]. Briefly, the reads from the 454 sequencing run were decoded by requiring a perfect match to the sample barcode and were subsequently trimmed. Next, the collection was trimmed and quality filtered by requiring a 95% match to the LTR primer, and 100% match to the flanking LTR region. Finally, only those sequences were considered as authentic integration sites that began within 3 bp of the LTR end and showed best unique alignments to the human genome by BLAT (hg18, version 36.1, >98% match score). The HIV-1 integration sites were matched to computationally-generated random control sites. Fisher's exact test was used to compare the distribution of integration sites with respective matched random controls (MRCs). The frequency of integration of the indicated drug-treated sample with the diluent control (DMSO) sample was compared using Fisher's exact test. Statistical analysis was performed using R (http://www.r-project.org).

The association of HIV-1 integration sites to the genomic features was performed as described previously [20], [77]. Briefly, the relationship between the integration site frequencies relative to the matched random controls for each of the annotated genomic feature was quantified using receiver operator characteristic (ROC) curve area method. The ROC value of each comparison is represented as a tile in the heatmap. Statistical methods and tests to determine whether the ROC areas calculated were significantly different from one another or from 0.5 (matched random controls) have been described previously [78].

### RNA isolation and analyses

Total cellular RNA was extracted using RNeasy Mini Kit (Qiagen) and subjected to on-column DNase digestion following the manufacturer's protocol. Virion RNA was isolated from pelleted virions using TRIzol LS (Invitrogen), treated with TURBO DNase (Ambion), and subjected to acid phenol extraction (Life Technologies) followed by ethanol precipitation. Complementary DNA (cDNA) was synthesized using Omniscript reverse transcriptase (RT) kit (Qiagen), random hexamers and 100 ng of RNAs following the manufacturer's protocol. 10% of the RT reaction was used for SYBR-green (iQ SYBR Green Supermix; Biorad) based qPCR using previously described [79] HIV-1 gag and gapdh: gag forward 5′-GTAAGAAAAAGGCACAGCAAGCAGC, gag reverse 5′-CATTTGCCCCTGGAGGTTCTG, gapdh forward 5′-CATCAATGACCCCTTCATTGAC, and gapdh reverse 5′-CGCCCCACTTGATTTTGGA. Standard curves to calculate absolute gag RNA copy numbers were generated using HIV-1 provirus plasmid (pNL4-3) in the range of 10^2^ to 10^8^ copies. qPCR reactions using cellular RNA were normalized to GAPDH and relative quantification analysis was performed using the 2^−ΔΔCT^ method [73].

### Virion protein processing

HEK293T cells (2×10^6^ cells/100 mm culture dish in 10 ml of complete medium) were transfected with HIV-1 provirus (pNL4-3) and cultured for 24 h to ensure expression of the provirus as described above. Next, the cells were treated with the indicated inhibitor or diluent control (DMSO) for 1 h. The culture supernatant was replaced with fresh complete medium containing either the inhibitor or DMSO, and cells were then cultured for another 24 h to allow for the production of HIV-1 particles in the presence of the inhibitor or diluent control. The virus containing cell-free supernatant was collected, filtered through 0.45 µm filter, and the amounts of viral particles produced was measured by HIV-1 Gag p24 ELISA (ZeptoMetrix) following manufacturer's protocol. Equivalent amounts of virions (∼5 µg p24 based on HIV-1 p24 ELISA) were concentrated over a 25% sucrose cushion by ultra-centrifugation at 28,000 rpm for 2 h at 4°C in SW41 rotor (Beckman). Pelleted virions were lysed in 100 µL RIPA buffer containing 50 mM Tris (pH 8.0), 150 mM NaCl, 0.1% SDS, 1% Triton-X, 1% deoxycholic acid, and 2 mM PMSF. Indicated amounts of concentrated virions were resolved by SDS-PAGE (4–12%, Invitrogen) and immunoblotted.

### Virion-associated IN cross-linking assay

The IN cross-linking assay was performed as described previously [41]. Briefly, concentrated virions equivalent to 50 ng of HIV-1 p24 were cross-linked with 50 µM bis(sulfosuccinimidyl)suberate (BS^3^) cross-linking reagent (Thermo Scientific). Subsequent cross-linked reaction products were quenched and resolved by SDS-PAGE (4–12%, Invitrogen) and immunoblotted.

### Virion-associated Reverse Transcriptase (RT) assay

Virions generated in the presence of inhibitor or diluent control (DMSO) was concentrated over 25% sucrose cushion. The virion-associated RT activity assay was performed as described previously [80], [81] with minor changes. Briefly, equivalent amounts of concentrated virions (based on HIV-1 p24 ELISA) were incubated with RT buffer, containing 50 mM Tris (pH 7.8), 75 mM KCL, 5 mM MgCl_2_, 2 mM DTT, 0.05% Nonidet P-40 (v/v), 5 µg/ml Poly r(A). Poly d(T)_12–18_, and 10 µCi of α-^32^P dTTP, in 35 µl final volume. The reactions were incubated at room temperature for 3 h. Duplicate 10 µl of the final reactions were blotted onto a 96-well DEAE Filtermat paper (Wallac-Perkin Elmer), dried, washed and processed as described previously.

### tRNA^Lys3^ primer extension assay

Total viral RNA, which serves as a source of tRNA^Lys3^ primer annealed to the viral RNA template, was extracted as described above. The relative amount of viral RNA template from the virions produced in the presence of inhibitor or diluent control (DMSO) was determined using quantitative PCR as described above. The initiation of reverse transcription from total viral RNA was measured using an *in vitro* HIV-1 reverse transcription reaction, which extends tRNA^Lys3^ primer on viral RNA template by +6-nt, as described previously [82], [83] with minor modifications. Briefly, 10^7^ copies of total viral RNA were incubated with 50 ng of purified HIV-1 RT (NIH Catalog # 3555) at 37°C for 15 min in 20 µl of RT buffer containing 50 mM Tris-HCl (pH 7.5), 60 mM KCl, 3 mM MgCl_2_, 5 mM DTT, 10 U of RNaseOut (Invitrogen), 200 µM dCTP, 200 µM dTTP, 50 µM ddATP and 5 µCi of [α-^32^P] dGTP. The reaction was stopped by adding 180 µl of termination buffer (100 µl isopropanol, 10 µl NaOAc, 70 µl ddH2O and 1 µg linear acrylamide) and incubating the samples at −80°C for 1 h to precipitate reverse transcription product. The final reaction products were resolved by PAGE (6% polyacrylamide, 7M urea) and detected by the phosphorimager analysis.

### Virion RNA packaging

HEK293T cells (2×10^6^ cells/100 mm culture dish in 10 ml of complete medium) were transfected with HIV-1 provirus (pNL4-3) and cultured for 24 h to ensure expression of the provirus as described above. Next, the cells were treated with the indicated inhibitor or diluent control (DMSO) for 1 h. The culture supernatant was replaced with fresh complete medium containing either the inhibitor or DMSO, and cells were then cultured for another 24 h to allow for the production of HIV-1 particles in the presence of the inhibitor or diluent control. The virus containing cell-free supernatant was collected, filtered through a 0.45 µm filter, and concentrated over a 25% sucrose cushion by ultra-centrifugation at 28,000 rpm for 2 h at 4°C in SW41 rotor (Beckman). In parallel, aliquots of cell-free supernatant were used to measure the amount of viral particles produced using HIV-1 Gag p24 ELISA (ZeptoMetrix) following manufacturer's protocol. RNA was isolated from the pelleted virions and subsequent cDNA preparations were used for gag quantitative PCR as described above. Relative virion RNA packaging was calculated by normalizing virion Gag RNA copy numbers to Gag p24.

### Viral entry assay

The viral entry assay [84] with some modifications was used to measure the incoming viral genomic RNA upon viral fusion and entry. Briefly, HEK293T cells (2×10^5^ cells/well of a 6-well plate in 2 ml of complete medium) were infected with DNase (60 U/ml, Ambion)-treated HIV-1 virions equivalent to 0.5×10^7^ viral RNA copies for 30 min at 4°C. The infected cultures were then incubated at 37°C and 5% CO_2_ for 1 h. Next, infected cells were harvested and total cellular RNA was isolated using RNeasy mini kit (Qiagen) and subjected to on-column DNase digestion following the manufacturer's protocol. cDNA synthesis and qPCR using HIV-1 gag and gapdh primers were performed as described above. The Gag qPCRs were normalized to GAPDH as described above.

### Viral genomic RNA stability assay

The stability of incoming viral genomic RNA was measured as described previously [84] with minor modifications. Briefly, HEK293T cells (2×10^5^ cells/well of a 6-well plate in 2 ml of complete medium) were infected with DNase (60 U/ml, Ambion)-treated HIV-1 virions equivalent to 2 µg p24 based on HIV-1 p24 ELISA for 30 min at 4°C. The infected cultures were then incubated at 37°C and 5% CO_2_. Infected cells were harvested at the indicated times post-infection and total cellular RNA was isolated using RNeasy mini kit (Qiagen) and subjected to on-column DNase digestion following the manufacturer's protocol. cDNA synthesis and qPCR using HIV-1 gag and gapdh primers were performed as described above. The Gag qPCRs were normalized to GAPDH as described above. In order to avoid any contamination of leftover HIV-1 proviral plasmid DNA from transfections to produce cell-free virions, which might interfere with gag qPCRs, 1 µM NVP was added to the cell culture. Briefly, cells were pre-incubated with 1 µM NVP for 2 h and subsequent viral infections were performed in the presence NVP. The cells were cultured in the presence of NVP for indicated times before analysis.

## Supporting Information

Figure S1 **Synthesis of racemic KF115 (A) and KF116 (B).**
(PDF)

Figure S2 ***In vitro*** **activities of KF115 and KF116.** HTRF-based assays were performed to monitor KF115 and KF116 activities for promoting aberrant IN multimerization and inhibiting IN-LEDGF/p75 binding. (**A**) Dose dependent effects of KF115 on promoting aberrant IN multimerization. (**B**) Dose dependent effects of KF115 on IN-LEDGF/p75 binding. (**C**) Dose dependent effects of KF116 on promoting aberrant IN multimerization. (**D**) Dose dependent effects of KF116 on IN-LEDGF/p75 binding. Bars represent mean ± SD (*n* = 3). The results are summarized in Table 1.
(PDF)

Figure S3 **KF116 promotes allosteric IN multimerization of A128T IN** ***in vitro*** **and impairs A128T HIV-1_NL4-3_ replication in infected cells.** An overlay of crystal structures of WT and A128T IN CCDs bound to BI-1001 (**A**) or KF116 (**B**). (A): The sidechain of Ala-128 and its corresponding BI-1001 molecule are colored orange, whereas Thr-128 and its corresponding BI-1001 molecule are colored gray. (B): The sidechain of Ala-128 and its corresponding KF116 molecule are colored magenta, whereas Thr-128 and its corresponding KF116 molecule are colored gray. The hydrogen bonds between the inhibitors and IN subunits are shown by black dashed lines. Side chains of HIV-1 IN residues T125 in subunit 1, and E170, H171 and T174 in subunit 2 are shown. (**C**) Effects of BI-1001 or KF116 on A128T HIV-1 infectivity. HEK293T cells were transfected with HIV-1 provirus bearing a substitution in the IN gene (pNL4-3_A128T_). A128T HIV-1 particles were produced in the presence or absence of the indicated inhibitors. TZM-bl cells were infected with cell-free A128T virus equivalent to 4 ng of HIV-1 Gag p24 and luciferase assay was performed 48 hour post-infection. The luciferase signal obtained for the non-treated (DMSO) control was set to 100%. Bars represent mean ± SD (*n* = 3).
(PDF)

Figure S4 **KF116 does not affect virus production or viral protein processing.** HEK293T cells were transfected with HIV-1 provirus (pNL4-3). HIV-1 particles were produced in the presence of DMSO, 1 µM KF116, or 1 µM SQV. (**A**) Virus-containing cell-free supernatant were harvested and cell-free Gag was measured by HIV-1 Gag p24 ELISA. Bar graph indicates HIV-1 Gag p24 production relative to non-treated (DMSO) sample. Bars represent mean ± SD (*n* = 3). (**B**) Virus-containing cell-free supernatant equivalent to 5 µg HIV-1 Gag p24 were subjected to ultra-centrifugation and pelleted virions were detergent-lysed. Indicated amounts of pelleted virions were subjected to SDS-PAGE and immunoblotted with HIV-1 Gag, RT and IN antibodies.
(PDF)

Figure S5 **KF116 does not affect virion RNA packaging.** HIV-1 virions were produced in HEK293T cells in the presence of DMSO or 1 µM KF116, cell-free virions were harvested, concentrated by ultra-centrifugation, RNA was isolated from the pelleted virions, and subsequent cDNA preparations were used for Gag quantitative PCR. In parallel, aliquots of cell-free virus-containing supernatant were used to measure the amount of viral particles produced using HIV-1 Gag p24 ELISA. Virion RNA packaging was calculated by normalizing virion Gag RNA copy numbers to Gag p24. Bar graph indicates virion RNA packaging relative to non-treated (DMSO) sample. Bars represent mean ± SD (*n* = 3).
(PDF)

Figure S6 **KF116 does not affect initiation of reverse transcription or virion-associated reverse transcriptase (RT) activity.** (**A**) HIV-1 virions were produced in HEK293T cells in the presence of DMSO or 1 µM KF116 and total viral RNA, which serves as a source of tRNA^Lys3^ primer annealed to the viral RNA template, was extracted. The initiation of reverse transcription from total viral RNA was measured using an *in vitro* HIV-1 reverse transcription reaction, which extends tRNA^Lys3^ primer on viral RNA template by +6-nt. The final reaction products were resolved by PAGE and detected by phosphorimager analysis. (*Upper panel*) A representative image of the 6-nt extension products is shown. (*Lower panel*) The bar graph represents quantification of bands from the upper panel using ImageJ software. The signal intensity obtained for the non-treated (DMSO) sample was set to 100%. Bars represent mean ± SD (*n* = 4). (**B**) HIV-1 virions were produced in HEK293T cells in the presence of 1 µM KF116 or DMSO control. Cell-free virions were harvested and concentrated by ultra-centrifugation. Equivalent amounts of concentrated virions, based on HIV-1 p24 ELISA, were analyzed for virion-associated RT activity. Bar graphs indicate RT activity relative to non-treated (DMSO) control. Bars represent mean ± SD (*n* = 3).
(PDF)

Figure S7 **KF116 does not affect viral entry in the target cells.** VSV-G pseudotyped HIV-1 virions were produced in the presence of 1 µM KF116 or DMSO control. HEK293T cells were infected with DNase-treated virions equivalent to 0.5×10^7^ viral RNA copies. Cells were harvested 1 h post-infection, total cellular RNA was harvested, and subsequent cDNA preparations were used for Gag or GAPDH quantitative PCR. The incoming viral genomic RNA in target cells was calculated by normalizing Gag RNA to GAPDH RNA. Bar graphs indicate incoming viral genomic RNA relative to non-treated (DMSO) control. Bars represent mean ± SD (*n* = 3).
(PDF)

Figure S8 **Effects of 10 µM GS-B or KF116 on LEDGF/p75-dependent targeting of HIV-1 integration site distribution on chromatin.** 10 µM GS-B or 10 µM KF116 was added directly to the target cells and the cells were infected with untreated virions. (**A**) HIV-1 integration frequencies in RefSeq and known genes, comparing effects of KF116 and GS-B on LEDGF/p75-dependent targeting of HIV-1 integration. All the samples differed significantly from their respective matched random controls (using Fisher's exact test, *P<0.001*). Significant deviation from the control non-treated (DMSO) sample was calculated using Fisher's exact test and is denoted by asterisks (*** *P<0.001*). (**B**) Heatmap summarizing HIV-1 integration frequencies relative to the genomic features. The columns depict HIV-1 integration site data sets for the indicated drug treatments. The rows depict the analyzed genomic features and the base pair values shown in the rows indicate the size of a given genomic interval used for the analysis. The relationship between HIV-1 integration site frequencies relative to matched random controls for each of the genomic feature was quantified using the receiver operator characteristic (ROC) curve area method. The color key shows enrichment (indicated in red) or depletion (indicated in blue) of a given genomic feature near integration sites. *P* values were calculated for the individual integration site data sets compared to the matched random controls, ***P<0.001; **P<0.01; *P<0.05.
(PDF)

Table S1 **X-ray crystal structure data collection and refinements.**
(PDF)

Text S1 **Synthesis of (±)KF115 and (±)KF116.**
(PDF)
