# Supplementary figures and images for: A New Class of Multimerization Selective Inhibitors of HIV-1 Integrase
Source: PLoS Pathog. 2014 May 29;10(5):e1004171. doi: 10.1371/journal.ppat.1004171 (PMC4038613; doi:10.1371/journal.ppat.1004171)

**Figure S1.**

### A. Synthesis of (±)KF115

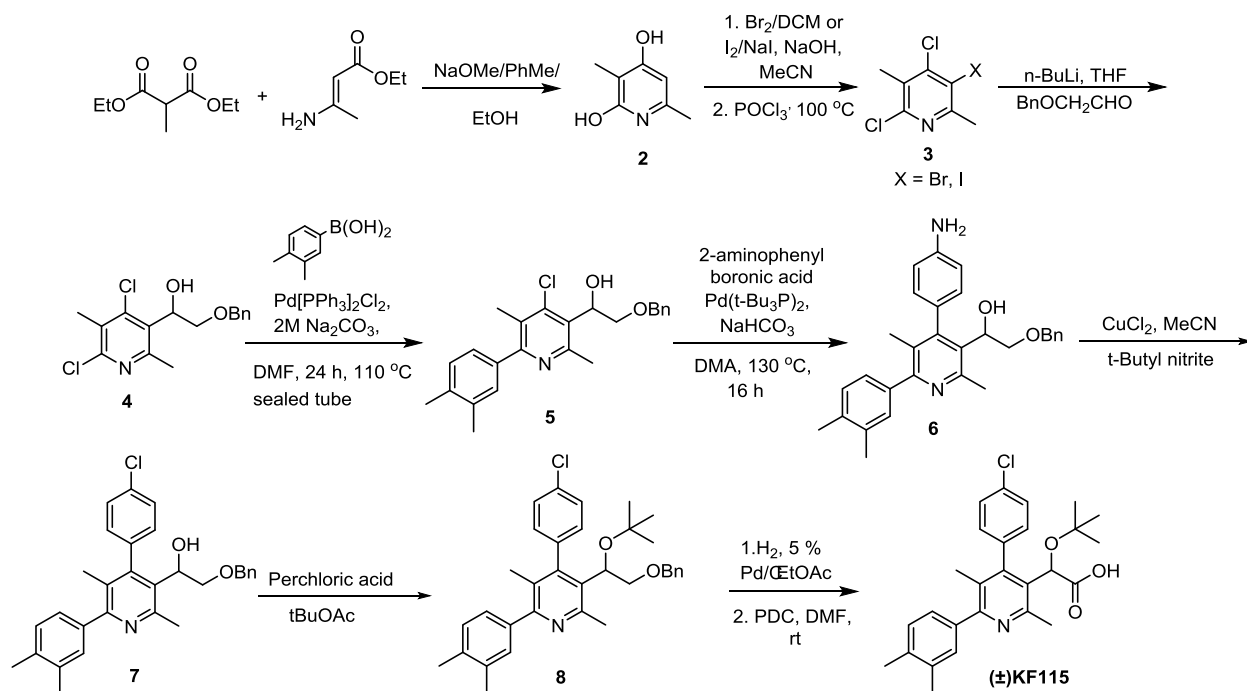

### B. Synthesis of (±)KF116

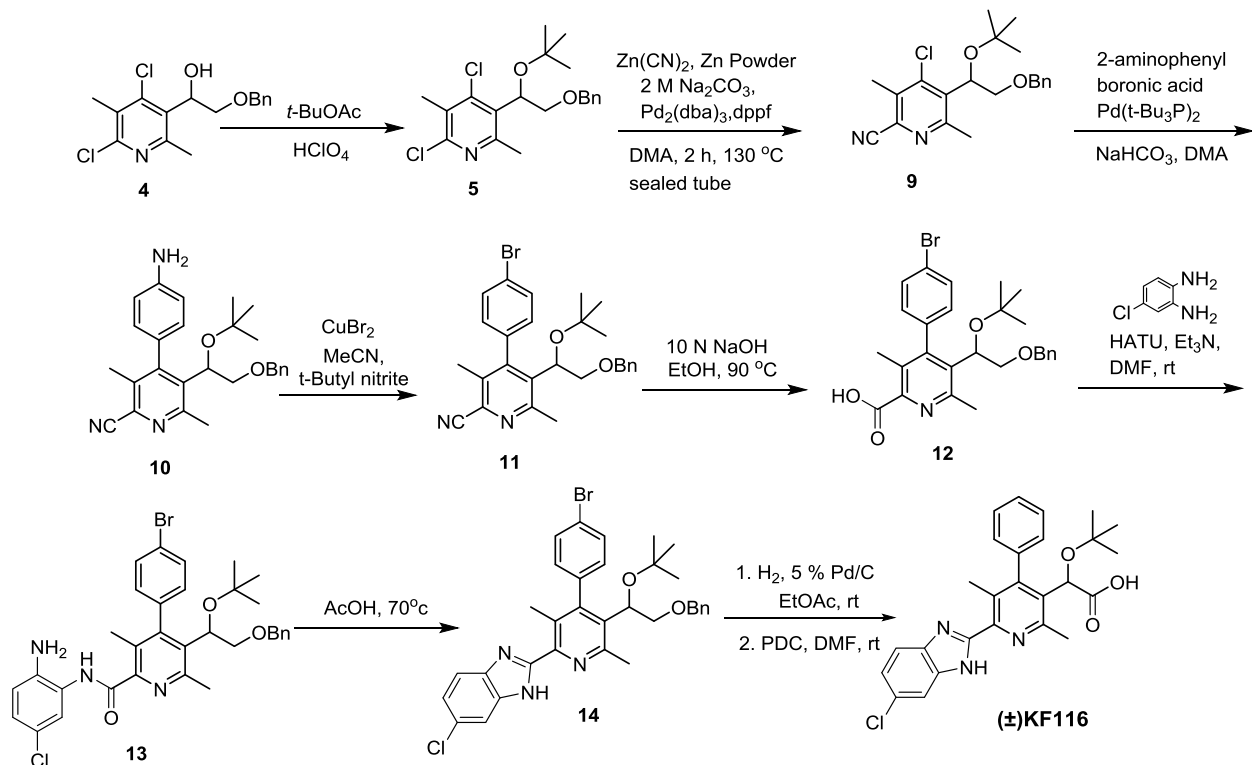

Supplement: Figure S1 — Synthesis of racemic KF115 (A) and KF116 (B). (PDF) [file ppat.1004171.s001.pdf]

**Figure S2**

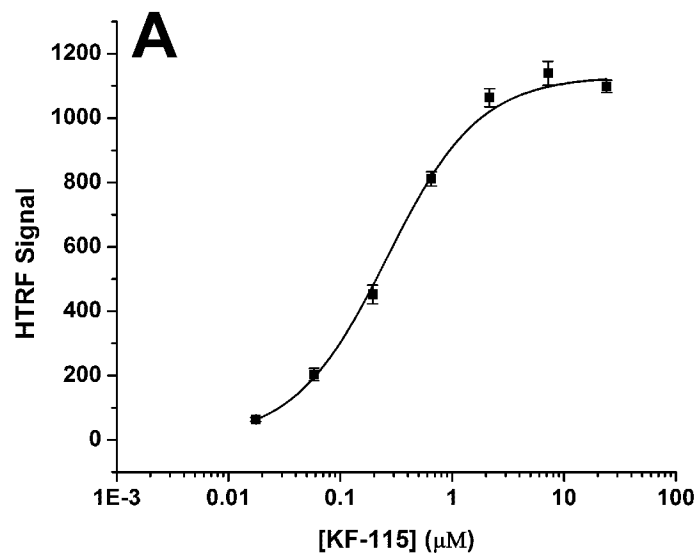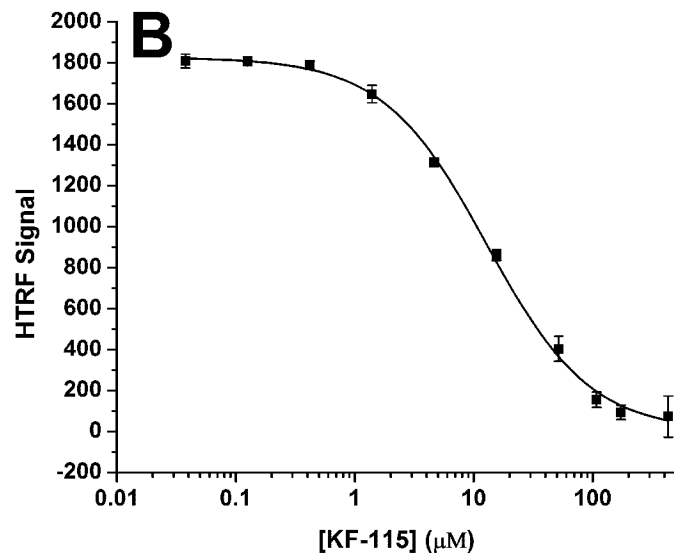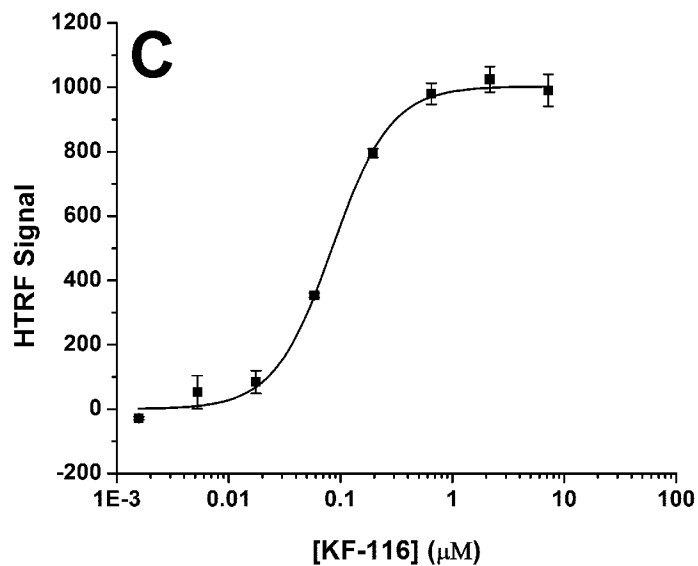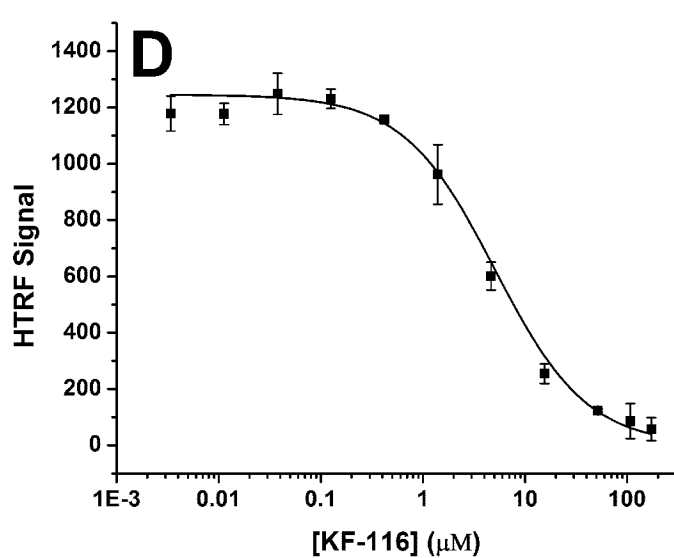

Supplement: Figure S2 — In vitro activities of KF115 and KF116. HTRF-based assays were performed to monitor KF115 and KF116 activities for promoting aberrant IN multimerization and inhibiting IN-LEDGF/p75 binding. (A) Dose dependent effects of KF115 on promoting aberrant IN multimerization. (B) Dose dependent effects of KF115 on IN-LEDGF/p75 binding. (C) Dose dependent effects of KF116 on promoting aberrant IN multimerization. (D) Dose dependent effects of KF116 on IN-LEDGF/p75 binding. Bars represent mean ± SD (n = 3). The results are summarized in Table 1. (PDF) [file ppat.1004171.s002.pdf]

Figure S3.

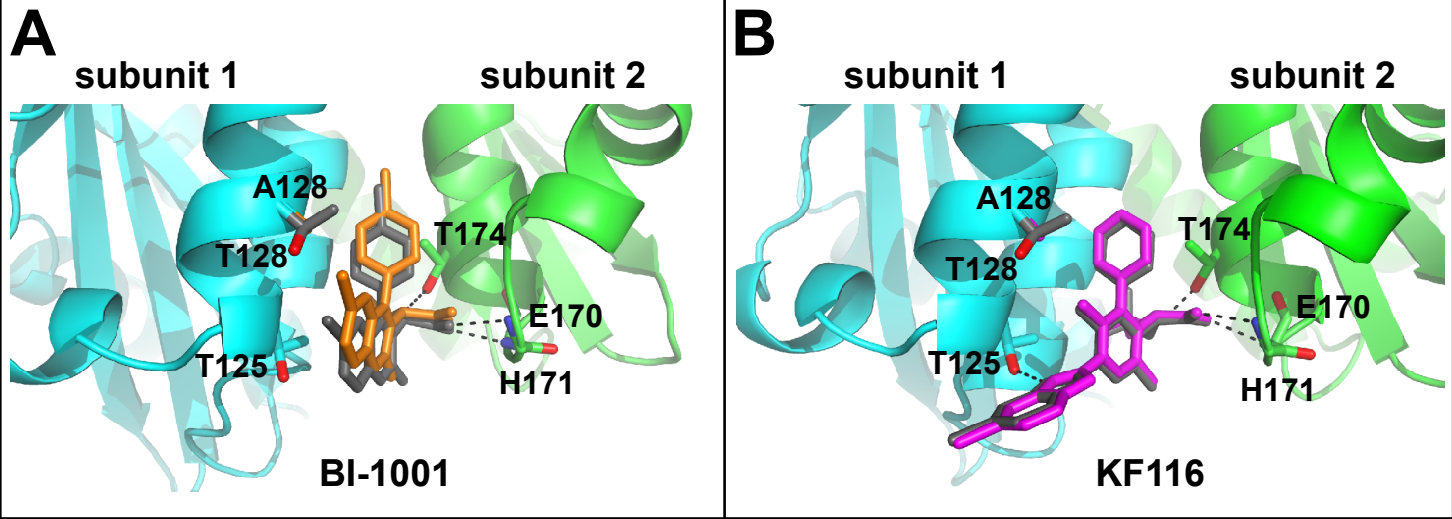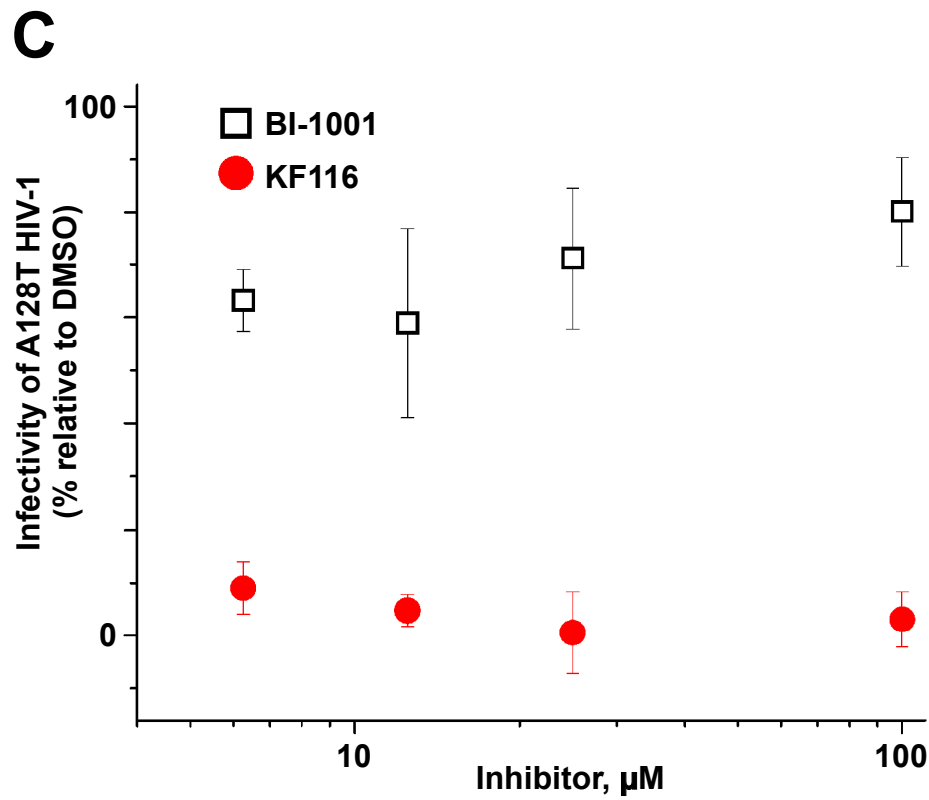

Supplement: Figure S3 — KF116 promotes allosteric IN multimerization of A128T IN in vitro and impairs A128T HIV-1NL4-3 replication in infected cells. An overlay of crystal structures of WT and A128T IN CCDs bound to BI-1001 (A) or KF116 (B). (A): The sidechain of Ala-128 and its corresponding BI-1001 molecule are colored orange, whereas Thr-128 and its corresponding BI-1001 molecule are colored gray. (B): The sidechain of Ala-128 and its corresponding KF116 molecule are colored magenta, whereas Thr-128 and its corresponding KF116 molecule are colored gray. The hydrogen bonds between the inhibitors and IN subunits are shown by black dashed lines. Side chains of HIV-1 IN residues T125 in subunit 1, and E170, H171 and T174 in subunit 2 are shown. (C) Effects of BI-1001 or KF116 on A128T HIV-1 infectivity. HEK293T cells were transfected with HIV-1 provirus bearing a substitution in the IN gene (pNL4-3A128T). A128T HIV-1 particles were produced in the presence or absence of the indicated inhibitors. TZM-bl cells were infected with cell-free A128T virus equivalent to 4 ng of HIV-1 Gag p24 and luciferase assay was performed 48 hour post-infection. The luciferase signal obtained for the non-treated (DMSO) control was set to 100%. Bars represent mean ± SD (n = 3). (PDF) [file ppat.1004171.s003.pdf]

**Figure S4.**

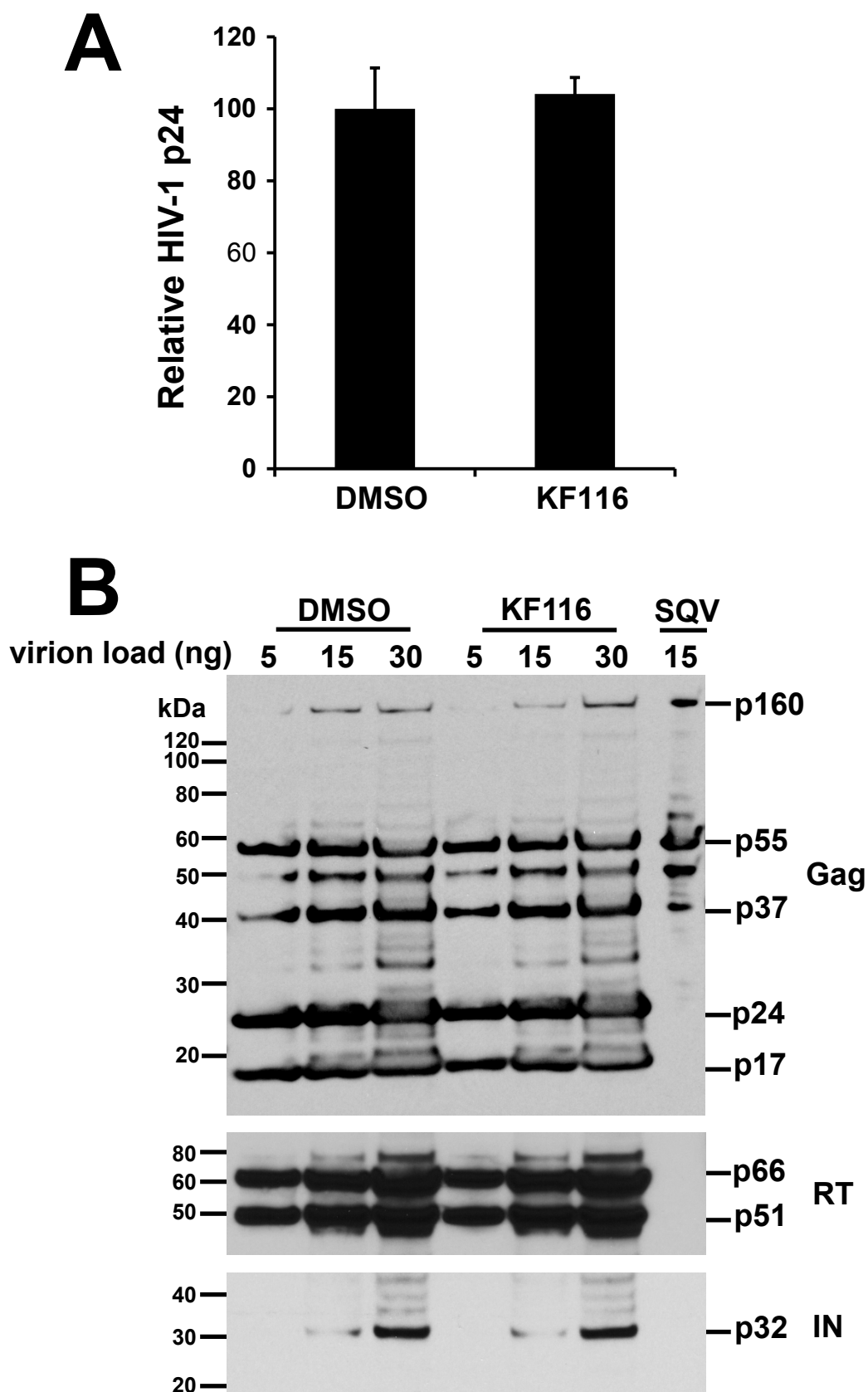

Supplement: Figure S4 — KF116 does not affect virus production or viral protein processing. HEK293T cells were transfected with HIV-1 provirus (pNL4-3). HIV-1 particles were produced in the presence of DMSO, 1 µM KF116, or 1 µM SQV. (A) Virus-containing cell-free supernatant were harvested and cell-free Gag was measured by HIV-1 Gag p24 ELISA. Bar graph indicates HIV-1 Gag p24 production relative to non-treated (DMSO) sample. Bars represent mean ± SD (n = 3). (B) Virus-containing cell-free supernatant equivalent to 5 µg HIV-1 Gag p24 were subjected to ultra-centrifugation and pelleted virions were detergent-lysed. Indicated amounts of pelleted virions were subjected to SDS-PAGE and immunoblotted with HIV-1 Gag, RT and IN antibodies. (PDF) [file ppat.1004171.s004.pdf]

**Figure S5.**

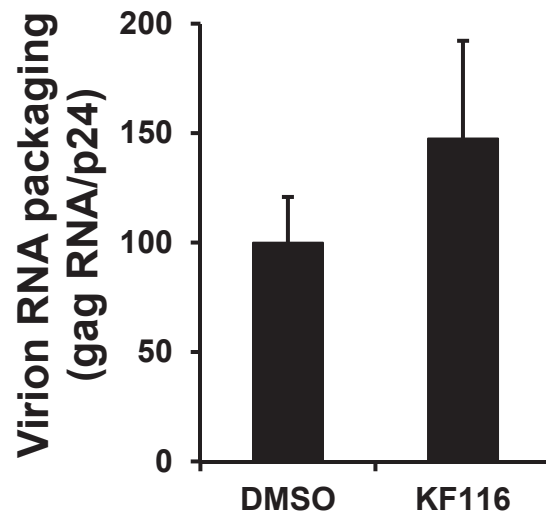

Supplement: Figure S5 — KF116 does not affect virion RNA packaging. HIV-1 virions were produced in HEK293T cells in the presence of DMSO or 1 µM KF116, cell-free virions were harvested, concentrated by ultra-centrifugation, RNA was isolated from the pelleted virions, and subsequent cDNA preparations were used for Gag quantitative PCR. In parallel, aliquots of cell-free virus-containing supernatant were used to measure the amount of viral particles produced using HIV-1 Gag p24 ELISA. Virion RNA packaging was calculated by normalizing virion Gag RNA copy numbers to Gag p24. Bar graph indicates virion RNA packaging relative to non-treated (DMSO) sample. Bars represent mean ± SD (n = 3). (PDF) [file ppat.1004171.s005.pdf]

**Figure S6.**

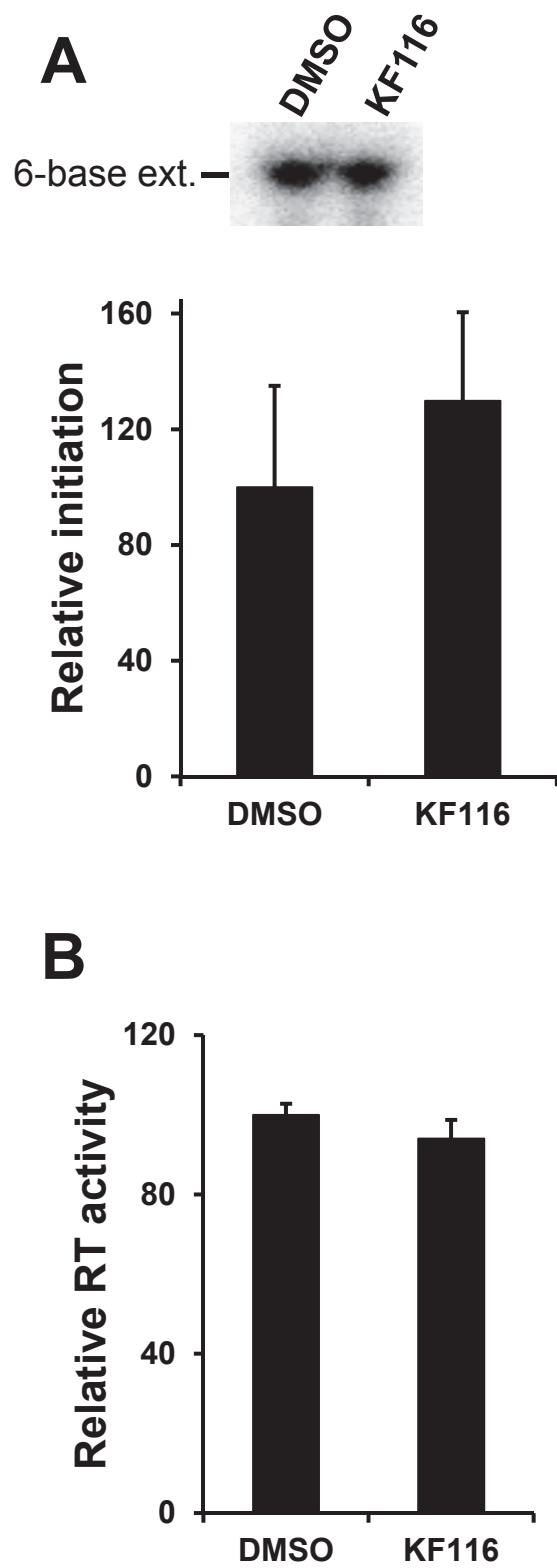

Supplement: Figure S6 — KF116 does not affect initiation of reverse transcription or virion-associated reverse transcriptase (RT) activity. (A) HIV-1 virions were produced in HEK293T cells in the presence of DMSO or 1 µM KF116 and total viral RNA, which serves as a source of tRNALys3 primer annealed to the viral RNA template, was extracted. The initiation of reverse transcription from total viral RNA was measured using an in vitro HIV-1 reverse transcription reaction, which extends tRNALys3 primer on viral RNA template by +6-nt. The final reaction products were resolved by PAGE and detected by phosphorimager analysis. (Upper panel) A representative image of the 6-nt extension products is shown. (Lower panel) The bar graph represents quantification of bands from the upper panel using ImageJ software. The signal intensity obtained for the non-treated (DMSO) sample was set to 100%. Bars represent mean ± SD (n = 4). (B) HIV-1 virions were produced in HEK293T cells in the presence of 1 µM KF116 or DMSO control. Cell-free virions were harvested and concentrated by ultra-centrifugation. Equivalent amounts of concentrated virions, based on HIV-1 p24 ELISA, were analyzed for virion-associated RT activity. Bar graphs indicate RT activity relative to non-treated (DMSO) control. Bars represent mean ± SD (n = 3). (PDF) [file ppat.1004171.s006.pdf]

**Figure S7.**

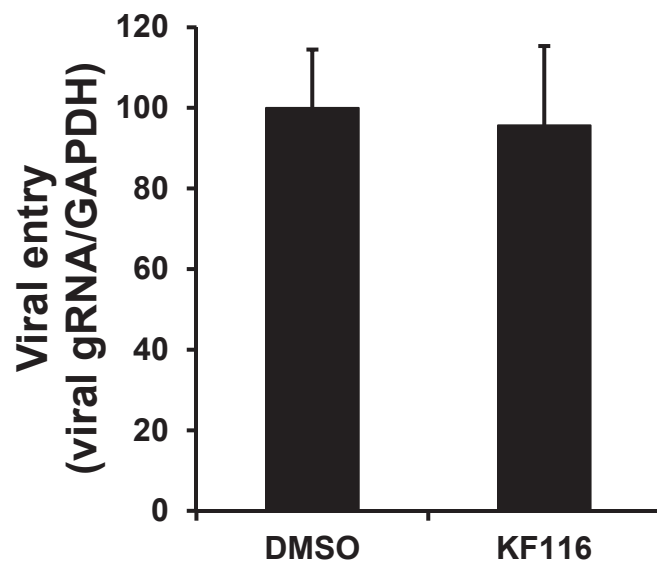

Supplement: Figure S7 — KF116 does not affect viral entry in the target cells. VSV-G pseudotyped HIV-1 virions were produced in the presence of 1 µM KF116 or DMSO control. HEK293T cells were infected with DNase-treated virions equivalent to 0.5×10^7^ viral RNA copies. Cells were harvested 1 h post-infection, total cellular RNA was harvested, and subsequent cDNA preparations were used for Gag or GAPDH quantitative PCR. The incoming viral genomic RNA in target cells was calculated by normalizing Gag RNA to GAPDH RNA. Bar graphs indicate incoming viral genomic RNA relative to non-treated (DMSO) control. Bars represent mean ± SD (n = 3). (PDF) [file ppat.1004171.s007.pdf]
