# Supplementary material for: A New Class of Multimerization Selective Inhibitors of HIV-1 Integrase
Source: PLoS Pathog. 2014 May 29;10(5):e1004171. doi: 10.1371/journal.ppat.1004171 (PMC4038613; doi:10.1371/journal.ppat.1004171)
Supplement: Figure S8 — Effects of 10 µM GS-B or KF116 on LEDGF/p75-dependent targeting of HIV-1 integration site distribution on chromatin. 10 µM GS-B or 10 µM KF116 was added directly to the target cells and the cells were infected with untreated virions. (A) HIV-1 integration frequencies in RefSeq and known genes, comparing effects of KF116 and GS-B on LEDGF/p75-dependent targeting of HIV-1 integration. All the samples differed significantly from their respective matched random controls (using Fisher's exact test, P<0.001). Significant deviation from the control non-treated (DMSO) sample was calculated using Fisher's exact test and is denoted by asterisks (*** P<0.001). (B) Heatmap summarizing HIV-1 integration frequencies relative to the genomic features. The columns depict HIV-1 integration site data sets for the indicated drug treatments. The rows depict the analyzed genomic features and the base pair values shown in the rows indicate the size of a given genomic interval used for the analysis. The relationship between HIV-1 integration site frequencies relative to matched random controls for each of the genomic feature was quantified using the receiver operator characteristic (ROC) curve area method. The color key shows enrichment (indicated in red) or depletion (indicated in blue) of a given genomic feature near integration sites. P values were calculated for the individual integration site data sets compared to the matched random controls, ***P<0.001; **P<0.01; *P<0.05. (PDF) [file ppat.1004171.s008.pdf]

Figure S8A

Treatment	Number of sites	% in RefSeq	% in known genes
DMSO	2141	81.83	83.61
KF116 target cells (10 μM)	2097	83.17	84.31
GS-B target cells (10 μM)	721	74.76***	77.39***

Figure S8B

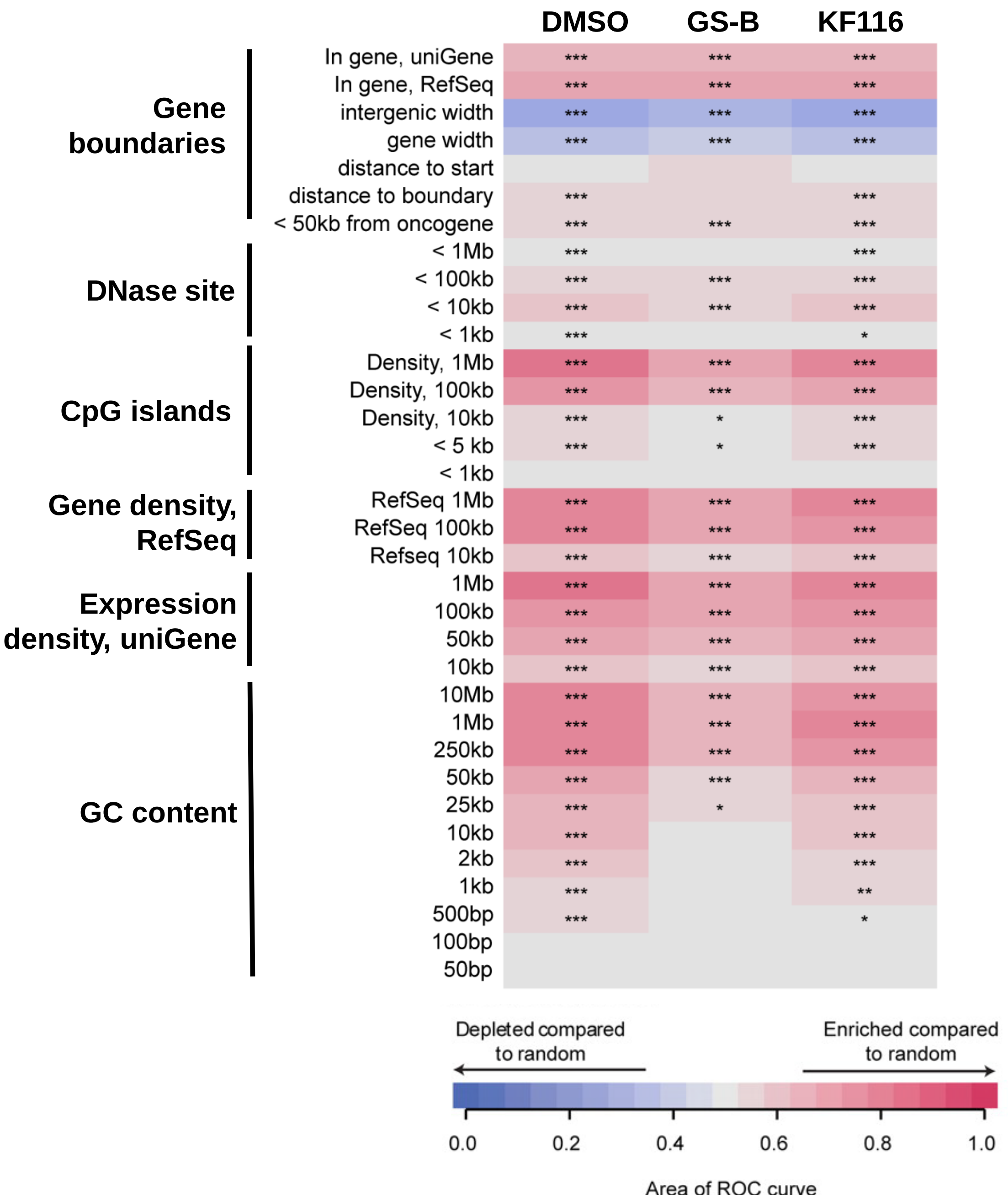
DMSO
GS-B
KF116
Gene boundaries
In gene, uniGene
In gene, RefSeq
intergenic width
gene width
distance to start
distance to boundary
< 50kb from oncogene
DNase site
< 1Mb
< 100kb
< 10kb
< 1kb
CpG islands
Density, 1Mb
Density, 100kb
Density, 10kb
< 5 kb
< 1kb
Gene density, RefSeq
RefSeq 1Mb
RefSeq 100kb
Refseq 10kb
Expression density, uniGene
1Mb
100kb
50kb
10kb
GC content
10Mb
1Mb
250kb
50kb
25kb
10kb
2kb
1kb
500bp
100bp
50bp
Depleted compared to random
Enriched compared to random
0.0
0.2
0.4
0.6
0.8
1.0
Area of ROC curve
