# Supplementary material for: A New Class of Multimerization Selective Inhibitors of HIV-1 Integrase
Source: PLoS Pathog. 2014 May 29;10(5):e1004171. doi: 10.1371/journal.ppat.1004171 (PMC4038613; doi:10.1371/journal.ppat.1004171)
Supplement: Table S1 — X-ray crystal structure data collection and refinements. (PDF) [file ppat.1004171.s009.pdf]

Table S1. X-ray crystal structure data collection and refinements

**Complex**	KF115 + HIV-1 CCD F185K	KF116 + HIV-1 CCD F185K	KF116 + HIV-1 IN CCD F185K/A128T
**Data Collection**			
Wavelength (Å)	1.541	1.541	1.541
Space group	P3121	P3121	P3121
Unit-cell parameters (Å, °)	a=b = 72.2, c=65.2 α =β =90, γ = 120	a=b = 71.7, c=65.9 α =β =90, γ = 120	a=b = 72.0, c=66.2 α =β =90, γ = 120
Molecules per A.S.U.	1	1	1
Resolution (Å)	2.04	2.20	2.37)
Completeness (%)	98.9	99.7	99.5
R_{merge} (%)	4.5 (47.7)	4.2 (58.4)	4.8 (55.8)
Redundancy	5.2 (5.2)	4.7 (4.7)	5.0 (5.0)
I/σ	45.3 (4.36)	39.6 (2.88)	45.2 (3.63)
**Structure Refinement**			
Resolution (Å)	2.05	2.24	2.37
R_{work} / R_{free} (%)	0.1838 / 0.2315	0.1751 (0.2588)	0.1851 / 0.2330
**R.M.S.D. from ideal values**			
Bond length (Å)	0.0184	0.0149	0.0159
Bond angle (°)	2.118	1.809	1.871
Average B factor	49.6	43.1	44.6
PDB #	**4O0J**	**4O55**	**4O5B**
