# Supplementary material for: A New Class of Multimerization Selective Inhibitors of HIV-1 Integrase
Source: PLoS Pathog. 2014 May 29;10(5):e1004171. doi: 10.1371/journal.ppat.1004171 (PMC4038613; doi:10.1371/journal.ppat.1004171)
Supplement: Text S1 — Synthesis of (±)KF115 and (±)KF116. (PDF) [file ppat.1004171.s010.pdf]

**Text S1. Synthesis of (±)KF115**

**3,6-dimethylpyridine-2,4-diol 2:** Dimethylmethylmalonate (1.89 g or 1.86 mL, 10.9 mmol) predissolved in anhydrous toluene (0.9 mL) was added to the sodium ethoxide (4.16 mL, 21 % W/W in EtOH) at room temperature. The resulting solution was stirred for 30 min and a solution of ethyl 3-aminocrotonate (1.37 g or 1.35 mL, 10.7 mmol) in anhydrous toluene (1.37 mL) was added. The mixture was stirred at reflux for 39 h, EtOH is removed under reduced pressure, the mixture was cooled and water (2 mL) is added. The mixture was stirred at 40 $^{o}$C for ~2 h, and then cooled to room temperature. The mixture was poured into a separating funnel and the phases were separated. The aqueous phase was washed with toluene (2 x 10 mL) and then adjusted to pH 5-6 with conc. HCl. The yellow solid was filtered and dried to afford dihydroxypyridine **2**. m.p., 270-72 $^{o}$C; $^{1}$H NMR (400 MHz, DMSO-$d_6$) δ 10.94 (br s, 1H), 9.95 (br s, 1H), 5.66 (s, 1H), 2.05 (s, 3H), 1.72 (s, 3H). $^{13}$C NMR δ (100 MHz, DMSO-$d_6$) δ 165.6, 163.8, 142.5, 103.9, 98.7, 19.2, 9.21.

**3-bromo-4,6-dichloro-2,5-dimethylpyridine 3:** Bromine (0.2 mL) pre-dissolved in DCM (2.5 mL) was added dropwise to dihydroxypyridine **2** (564 mg, 4.05 mmol) suspended in DCM (2 mL) at room temperature. The resulting mixture was stirred for 1 h and precipitated solid was filtered to give 5-bromo-3,6-dimethylpyridine-2,4-diol. HBr salt. The HBr. Salt (1.2 g, 4.0 mmol) was heated to reflux in $POCl_3$ (7 mL) for 17 h and cooled to room temperature. The excess $POCl_3$ was distilled off and the residue was poured onto ice. The mixture was adjusted to basic pH using 10 N NaOH and solid $NaHCO_3$. The mixture was filtered and filtrate was extracted with DCM (4 x 10 mL) and dried over sodium sulfate. Flash chromatography ($SiO_2$, 20% ethyl acetate in hexanes) afforded brominated pyridine **5.** $^{1}$H NMR (400 MHz, $CDCl_3$) δ 2.65 (s, 3H), 2.50 (s, 3H). $^{13}$C NMR δ (100 MHz, $CDCl_3$) δ 156.2, 149.3, 145.8, 129.7, 120.8, 25.8, 18.2.

**2-(benzyloxy)-1-(4,6-dichloro-2,5-dimethylpyridin-3-yl)ethanol 4:** *n*-BuLi (2.2 mL, 2.5 M in hexanes, 5.6 mmol) was added dropwise to the dichlorobromopyridine (1.27 g, 5.6 mmol) **3** dissolved in anhydrous THF (44 mL) under argon was at -78 $^{o}$C. After 1 h, benzyloxyacetaldehyde (925 mg, 6.16 mmol) was added drop wise and allowed to stir to 1 h at 78 $^{o}$C. The reaction was quenched with saturated $NH_4Cl$ and extracted with EtOAc (3 x 5 mL) dried over sodium sulfate and concentrated. Purification of the crude provided **4** (763 mg, 47 %) as colorless oil. $^{1}$H NMR (300 MHz, $CDCl_3$) δ 7.46 – 7.29 (m, 5H), 5.58 (dd, $J$ = 9.0, 3.9 Hz, 1H), 4.64 (s, 2H), 3.83 (dd, $J$ = 9.5 Hz, 1H), 3.63 (dd, $J$ = 9.9, 4.0 Hz, 1H), 2.68 (d, $J$ = 0.6 Hz, 3H), 2.47 (d, $J$ = 0.57 Hz, 3H).

**2-(benzyloxy)-1-(4-chloro-6-(3,4-dimethylphenyl)-2,5-dimethylpyridin-3-yl)ethanol 5:** To the dichloropyridine **4** (450 mg, 1.37 mmol) and 3,4- dimethylphenylboronic acid (248.2 mg, 1.65 mmol) in degassed DMF (4.6 mL) was added 2M $Na_2CO_3$ (1.76 mL, 3.53 mmol) followed by $PdCl_2(PPh_3)_2$ (96.7 mg, 0.13 mmol). The reaction mixture was again degassed by bubbling with

argon (10 min), the reaction vessel was sealed and heated at 110 $^{o}$C for 16 h. The cooled mixture was diluted with EtOAc, washed with water and brine, dried over $MgSO_4$ filtered and concentrated. Flash chromatography afforded **5** (295 mg, 54 %) as colorless oil. $^{1}$H NMR (300 MHz, $CDCl_3$) δ 7.45 – 7.30 (m, 5H), 7.28 (s, 1H), 7.19 (d, *J* = 15, 9, Hz, 2H), 5.62 (dd, *J* = 8.5, 3.5 Hz, 1H), 4.65 (dd, *J* = 18, 12 MHz, 2H), 3.91 (dd, *J* = 9.4 Hz, 1H), 3.81 (s, 1H), 3.69 (dd, *J* = 10.0, 3.8 Hz, 1H), 2.76 (s, 3H), 2.35 (s, 9H). $^{13}$C NMR (75 MHz, $CDCl_3$) δ 158.0, 155.4, 144.4, 137.6, 136.3, 136.1, 130.0, 129.1, 128.8, 128.2, 127.5, 127.5, 126.8, 126.2, 73.0, 71.6, 69.8, 23.7, 19.6, 19.4, 17.5.

**1-(4-(4-aminophenyl)-6-(3,4-dimethylphenyl)-2,5-dimethylpyridin-3-yl)-2-(benzyloxy)ethanol 6:** Chloropyridine **5** (280 mg, 0.71 mmol), 4-(4,4,5,5-tetramethyul-1,2,3-dioaborolan-2-yl)aniline (209.4 mg, 0.95 mmol), bis[tri-*t*-butlyphosphine]palladium(0) (36.2 mg, 0.07 mmol), $NaHCO_3$ (294.4 mg, 3.50 mmol) in anhydrous DMA (6.3 mL) and water (0.7 mL) was degassed by bubbling argon for 5 min. The mixture was heated to 130 $^{o}$C for 16 h in a sealed tube. The reaction mixture was cooled and diluted with EtOAc washed with water followed by brine. The organic layer is dried over sodium sulfate, filtered and concentrated. Purification of the crude provided **6** (184 mg, 57 %) as brown oil. $^{1}$H NMR (400 MHz, $CDCl_3$) δ 7.40 – 7.24 (m, 6H), 7.23 – 7.16 (m, 2H), 6.97 – 6.91 (m, 1H), 6.81 (dd, *J* = 8.1, 1.6 Hz, 1H), 6.78 – 6.69 (m, 2H), 5.01 (dd, *J* = 9.7, 3.5 Hz, 1H), 4.50 (q, *J* = 12.1 Hz, 2H), 3.82 (dd, *J* = 9.9 Hz, 2H), 3.49 (dd, *J* = 10.1, 3.5 Hz, 1H), 2.78 (s, 3H), 2.32 (s, 3H), 2.31 (s, 3H), 1.90 (s, 3H).

**2-(benzyloxy)-1-(4-(4-chlorophenyl)-6-(3,4-dimethylphenyl)-2,5-dimethylpyridin-3-yl)ethanol 7:** *t*-butyl nitrite (123.8 mg, 0.84 mmol) was added dropwise to a mixture of copper chloride (268.0 mg, 1.2 mmol) in anhydrous MeCN (8.3 mL) at 0 $^{o}$C under argon. A solution of aniline **6** (380 mg, 0.83 mmol) in anhydrous MeCN (8.3 mL) was added dropwise and the ice bath was removed after 15 min. After 16 h, the solvent was removed under reduced pressure and to residue was adsorbed onto silica gel and flash chromatography provided **7** (319 mg, 80%) as colorless oil. $^{1}$H NMR (300 MHz, $CDCl_3$) δ 7.44 (d, *J* = 8.7 Hz, 1H), 7.42-7.30 (m, 4H), 7.29-7.21 (m, 3H), 7.19 (s, 1H), 7.13 (d, J = 8.7 Hz, 1H), 6.95 (d, *J* = 7.8 Hz, 1H), 4.83 (d, *J* = 6.7 Hz, 1H), 4.49 (q, *J* = 12.0 Hz, 2H), 3.77 (dd, *J* = 9.7 Hz, 1H), 3.43 (dd, *J* = 6.9 Hz, 1H), 2.79 (s, 3H), 2.31 (s, 6H), 1.85 (s, 3H).

**3-(2-(benzyloxy)-1-(*tert*-butoxy)ethyl)-4-(4-chlorophenyl)-6-(3,4-dimethylphenyl)-2,5-dimethylpyridine 8:** The above chloride **7** (100 mg, 0.21 mmol**)** and *tert*-butyl acetate (4.2 mL, 0.05 M) were cooled in an ice bath and perchloric acid (0.59 mL, 0.009 mmol) was added rapidly. The flask is sealed and stirred for 4 h at 0-5 $^{o}$C. The reaction was quenched with saturated solution of sodium carbonate and the mixture is adjusted to pH 8-9 with solid sodium carbonate.

The mixture was filtered and washed with DCM. The organic layer was separated and aqueous layer was extracted with DCM dried over sodium sulfate, filtered and concentrated. Flash chromatography provided **8** (68 mg, 61 %**)** as colorless oil. $^1$H NMR (400 MHz, $CDCl_3$) δ 7.49 (d, *J* = 8 Hz, 1H), 7.34 (m, 5H), 7.27 – 7.14 (m, 5H), 6.98 (d, *J* = 8.0 Hz, 1H), 4.55 (s, 1H), 4.47 (q, *J* = 12.4 Hz, 2H), 3.74 (dd, *J* = 8 Hz, 1H), 3.54 (dd, *J* = 8, 4 Hz, 1H), 2.82 (s, 3H), 2.33 (s, 3H), 2.32 (s, 3H), 1.86 (s, 3H), 1.06 (s, 9H). $^{13}$C NMR (100 MHz, $CDCl_3$) δ 157.5, 155.6, 148.7, 138.9, 137.8, 136.7, 136.5, 134.0, 131.4, 130.8, 129.6, 129.4, 129.1, 128.7, 127.9, 127.9, 126.9, 126.0, 74.9, 73.3, 73.0, 28.9, 20.2, 19.9, 18.4.

**2-(*tert*-butoxy)-2-(4-(4-chlorophenyl)-6-(3,4-dimethylphenyl)-2,5-dimethylpyridin-3-yl)acetic acid (KF115):** To the solution of **8** (92 mg, 0.17 mmol) in EtOAc/MeOH (3:1, 9 mL) was added 5 % Pd/C (9.2 mg) and stirred at room temperature under balloon of hydrogen gas for 12 h. The reaction mixture was filtered over celite and washed it with EtOAc (3 x 5 mL). The filtrate was concentrated and purified through column chromatography to give mixture of unseparable alcohols which was taken further for next step. The mixture of alcohols (50 mg, 0.11 mmol) were treated with PDC (236.2 mg, 0.63 mmol) in DMF (0.23 mL) and was stirred for 16 h at room temperature. The mixture was quenched with cold water and extracted with ethyl acetate (3 x 5 mL). The combined organic phases were washed with brine, dried over $Na_2SO_4$, and concentrated under reduced pressure. The crude was purified through column chromatography, pure fractions were collected and concentrated to yield **(±) KF115** (6.1 mg, 12%) as an off-white solid: mp 148.2-151.6 °C ; $^1$H NMR (400 MHz, DMSO as mixture of atropisomers) δ 7.92 (dd *J* = 12, 8 MHz, 1H), 7.53 – 7.47 (m, 1H), 7.46-7.35 (m, 1H), 7.32-7.23 (m, 2H), 7.22-7.15 (m, 1H), 4.41 (s, 1H), 2.53 (s, 3H), 2.27 (s, 6H), 1.85 (s, 3H), 0.83 (s, 9H). HRMS-ESI *m/z* $(M\text{-}Cl + H)^+$ calcd for $C_{27}H_{30}ClNO_3$, 418.2382 found 418.2375.

**Synthesis of (±)KF116**

**3-(2-(benzyloxy)-1-(*tert*-butoxy)ethyl)-4,6-dichloro-2,5-dimethylpyridine 5:** **4** (1.4 g, 4.2 mmol**)** and *tert*-butyl acetate (84 mL, 0.05 M) was cooled in an ice bath and perchloric acid (12 mL, 0.19 mmol) added quickly. The flask is sealed and stirred for 4 h at 0-5 $^o$C. The reaction was quenched with saturated solution of sodium carbonate and the mixture was adjusted to pH 8-9 with solid sodium carbonate. The mixture was filtered and washed with DCM. The organic layer was separated and the aqueous layer was extracted with DCM dried over sodium sulfate, filtered and concentrated. Flash chromatography ($SiO_2$, 10 % EtOAc in hexanes) of the crude provided **5** (1.3 g, 79 %**)** as colorless oil. $^1$H NMR (300 MHz, $CDCl_3$) δ 7.43 – 7.14 (m, 5H), 5.51 – 5.36 (m,

1H), 4.59 (q, *J* = 12.2 Hz, 2H), 3.84 – 3.65 (m, 1H), 3.54 (dd, *J* = 10.4, 5.1 Hz, 1H), 2.69 (s, 3H), 2.47 (s, 3H), 1.17 (s, 9H).

**5-(2-(benzyloxy)-1-(*tert*-butoxy)ethyl)-3,6-dimethyl-4-phenylpicolinonitrile 9:** Dicholoropyrindine **5** (327 mg, 0.84 mmol), $Pd_2(dba)_3$ (15.4 mg, 0.016 mmol), dppf (18.4 mg, 0.03 mmol), Zn powder (6.54 mg, 0.10 mmol), Zinc cyanide (58.9 mg, 0.50 mmol), were taken in degassed anhydrous DMA (2.0 mL) and heated in a sealed tube for 130 $^{o}$C for 2 h. The mixture was then cooled to room temperature and quenched with water and extracted with EtOAc (3 x 5 mL). The combined organic layers were washed with brine and dried over sodium sulfate and concentrated. Flash chromatography ($SiO_2$, 13 % EtOAc in hexanes) provided **9 (**300 mg, 95%) as colorless oil. $^{1}$H NMR (400 MHz, $CDCl_3$) δ 7.55-7.49 (m, 3H), 7.37 – 7.22 (m, 4H), 7.22 – 7.07 (m, 3H), 6.99 (d, *J* = 6.9 Hz, 1H), 4.60 (t, *J* = 8 Hz, 1H), 4.39 (q, *J* = 12.3 Hz, 2H), 3.63 (dd, *J* = 8 Hz, 1H), 3.50 (dd, *J* = 10.2, 4.8 Hz, 1H), 2.78 (s, 3H), 2.12 (s, 3H), 0.98 (m, 9H). $^{13}$C NMR (100 MHz, $CDCl_3$) δ 158.3, 149.7, 138.0, 137.4, 136.4, 133.9, 131.7, 129.2, 129.0, 128.7, 128.4, 128.3, 127.5, 127.2, 116.9, 115.3, 74.9, 72.9, 72.2, 70.5, 28.2, 27.9, 24.4, 17.2.

**4-(4-aminophenyl)-5-(2-(benzyloxy)-1-(*tert*-butoxy)ethyl)-3,6-dimethylpicolinonitrile 10:** A solution of chloropyridine **9** (364 mg, 0.97 mmol), 4-(4,4,5,5-tetramethy-1,2,3-dioaborolan-2-yl)aniline (288.5 mg, 1.32 mmol), bis[tri-*t*-butlyphosphine]palladium(0) (49.9 mg, 0.09 mmol), $NaHCO_3$ (409.9 mg, 4.88 mmol) , anhydrous DMA (10.8 mL) and water (1.8 mL) was degassed by bubbling argon for 5 min. The mixture is heated to 130 $^{o}$C for 16 h in a sealed tube. The reaction mixture was cooled and diluted with EtOAc washed with water followed by brine. The organic layer was dried over sodium sulfate, filtered and concentrated. Flash chromatography (Deactivated $SiO_2$, 30% EtOAc in hexanes) provided aniline **10** (400 mg, 95%) as brown oil. $^{1}$H NMR (400 MHz, $CDCl_3$) δ 7.37–7.28 (m, 5H), 7.18-7.14 (m, 2H), 7.08 (d, *J* = 8.6 Hz, 1H), 6.98 (d, *J* = 9.6 Hz, 1H), 4.63 (t, *J* = 6.8 Hz, 1H), 4.44 (q, *J* = 12.2 Hz, 2H), 3.63 (dd, *J* = 8 Hz, 1H), 3.55 (dd, *J* = 10.2, 4.8 Hz, 1H), 2.77 (s, 3H), 2.11 (s, 1H), 1.01 (s, 9H).

**5-(2-(benzyloxy)-1-(*tert*-butoxy)ethyl)-4-(4-bromophenyl)-3,6-dimethylpicolinonitrile 11:** *t*-butyl nitrite (167.5 mg, 1.62 mmol) was added dropwise to a suspension of anhydrous copper (II) bromide (362.9 mg, 1.62 mmol) in anhydrous degassed MeCN (11 mL) at 0 $^{o}$C under argon. A solution of aniline **10** (48 mg, 1.13 mmol) in anhydrous MeCN (1.1 mL) was added dropwise and the ice bath was removed after 15 min. After 16 h, the solvent is removed under reduced pressure and the residue was adsorbed onto silicagel and purified by flash chromatography ($SiO_2$, 30 % EtOAc in hexanes) afforded **11** (420 mg, 85 %) as light yellow oil. $^{1}$H NMR (400 MHz, $CDCl_3$ observed as mixture of atropisomers, peaks reported for major isomer) δ

7.63-7.28 (m, 5H), 7.19-7.19 (m, 2H), 7.02 (d, *J* = 8.0 Hz, 1H), 6.89 (d, *J* = 8.0 Hz, 1H), 4.56 (m, 1H), 4.41 (q, *J* = 12.0 Hz, 2H), 3.61-3.54 (m, 2H), 2.77 (s, 3H), 2.11 (s, 3H), 1.01 (s, 9H).

**5-(2-(benzyloxy)-1-(*tert*-butoxy)ethyl)-4-(4-bromophenyl)-3,6-dimethylpicolinic acid 12:** The picolinonitrile **11** (420 mg, 0.85 mmol), EtOH (0.85 mL) and 10N aqueous NaOH (0.85 mL) were heated to 90 $^{o}$C for 16 h in a sealed round bottom flask. The reaction mixture was then cooled to room temperature; ethanol was removed under reduced pressure and neutralized with water and conc. HCl dropwise. The mixture was extracted with EtOAc and combined organic layers were dried over sodium sulfate and concentrated provided the crude acid **12** (325mg, 96%) as white solid. This was taken further to next step without further purification. $^{1}$H NMR (300 MHz, $CDCl_3$, observed as mixture of atropisomers, peaks reported for major isomer) δ 7.66 (d, *J* = 8.4 Hz, 1H), 7.54 (d, *J* = 8.4 Hz, 1H), 7.37 - 7.29 (m, 3H), 7.19-7.09 (m, 2H), 7.02 (d, *J* = 6.4 Hz, 1H), 6.86 (d, *J* = 7.8 Hz, 1H), 4.60–4.31 (m, 1H), 4.44 (q, J = 12.2 Hz, 2H), 3.66-3.45( m, 2H), 2.78 (s, 3H), 2.32 (s, 3H), 1.01 (s, 9H).

**N-(2-amino-5-chlorophenyl)-5-(2-(benzyloxy)-1-(*tert*-butoxy)ethyl)-4-(4-bromophenyl)-3,6-dimethylpicolinamide 13**: Triethylamine (191.2 mg, 1.87 mmol) was added to the solution of acid **12** (325 mg, 0.63 mmol), 4-chloro-*o*-phenylenediamine (68.5 mg, 0.63 mmol), HATU (289.3 mg, 0.76 mmol) dissolved in DMF (6.3 mL) at room temperature under agron. The mixture was stirred for 40 min at room temperature. The reaction mixture was diluted with EtOAc and water and the aqueous was extracted with EtOAc (3 x 5 mL). The combined organic layers were washed with brine, dried over sodium sulfate, filtered and concentrated. Flash chromatography (Deactivated $SiO_2$, 20 % EtOAc in hexanes) provided the amide **13** (107 mg, 26%) thick viscous oil. mp 60-62 $^{o}$C; $^{1}$H NMR (300 MHz, $CDCl_3$, observed as mixture of atropisomers, peaks reported for major isomer) 10.14 (brs, 1H), 7.66 (d, *J* = 7.5 Hz, 1H), 7.52 (dd, *J* = 8.4, 1.5 Hz, 1H),7.48 – 7.12 (m, 7H), 7.06 (d, *J* = 9 Hz, 1H), 6.88 – 6.81 (m, 2H), 4.62 - 4.46 (m, 2H), 4.38 (dd, *J* = 12.3, 4.6 Hz, 1H), 4.27 (brs, 2H), 3.65 (dd, *J* = 18.2, 9.0 Hz, 1H), 3.52 (dd, *J* = 10.2, 5.0 Hz, 1H), 2.83 (s, 3H), 2.32(s, 3H), 1.03 (s, 9H). HRMS-ESI *m/z* $(M+H)^+$ calcd for $C_{33}H_{35}BrClN_3O_3$, 636.1629 found 636.1614.

**2-(5-(2-(benzyloxy)-1-(*tert*-butoxy)ethyl)-4-(4-bromophenyl)-3,6-dimethylpyridin-2-yl)-6-chloro-1H-benzo[d]imidazole 14:** The amide **13** (107 mg, 0.17 mmol) was then taken in glacial acetic acid (2 mL) and heated to 70 $^{o}$C for 40 min. The solution was then concentrated under reduced pressure, diluted with EtOAc and washed with saturated aqueous $NaHCO_3$ and brine. The organic layer was dried ovr sodium sulfate and concentrated. Flash chromatography (Deactivated $SiO_2$, 20 % EtOAc in hexanes) afforded the benzimidazole **14** (65 mg, 62%) as yellow foamy solid. $^{1}$H NMR (300 MHz, $CDCl_3$, observed as mixture of atropisomers, peaks

reported for major isomer) 10.79 (brs, 1H), 7.66 (d, *J* = 9.9 Hz, 1H), 7.52 (d, *J* = 8.0 Hz, 1H),7.57 – 7.54 (m, 2H), 7.37 - 7.25 (m, 4H), 7.24 - 7.14 (m, 2H), 7.10 (d, *J* = 7.8 Hz, 1H), 6.90 (d, *J* = 7.5 Hz, 1H), 4.64 - 4.44 (m, 2H), 4.38 (dd, *J* = 12.3, 4.6 Hz, 1H), 3.68 (dd, *J* = 18.2, 9.0 Hz, 1H), 3.53 (dd, *J* = 10.2, 5.0 Hz, 1H), 2.84 (s, 3H), 2.53(s, 3H), 1.03 (s, 9H). HRMS-ESI *m/z* $(M+H)^+$ calcd for $C_{33}H_{33}BrClN_3O_3$, 618.1523 found 618.1528.

**2-(*tert*-butoxy)-2-(6-(6-chloro-1H-benzo[d]imidazol-2-yl)-2,5-dimethyl-4-phenylpyridin-3-yl)acetic acid (KF-116):** To the benzimidazole **14** (66 mg, **)** dissolved in EtOAc /MeOH ( 4 mL, 3:1) was added 5 % Pd/C (6.6 mg) and stirred under hydrogen balloon pressure for 16 h. The reaction mixture was filtered through celite washed with EtOAc (3 x 10 mL) and concentrated. The filtrate was concentrated and purified through column chromatography to give mixture of compounds (with halogen and without halogen) which was taken further for next step. The mixture of alcohols (30 mg, 0.05 mmol) was then treated with PDC (106.7 mg, 0.28 mmol) in DMF (0.16 mL) and stirred for 12 h at room temperature. The reaction mixture was then diluted with EtOAc and water, layers were separated and aqueous layer was saturated with NaCl and then extracted with EtOAc (3 x 5 mL). The combined organic layers were dried over sodium sulfate and concentrated. Preparative HPLC (conditions: XBridge™ Prep C18 OBD 5 µm reverse phase column, 19 x 150 mm; gradient; 0.1 % formic acid in MeOH and 0.1% formic acid as solvents 70 : 30; $t_R$ = 18.00 min) afforded (±) **KF116** as off-white solid. Analytical HPLC $t_R$ = 14.0 (column conditions: XBridge C18 5 µm ; 4.6 x 150 mm gradient (gradient; 0.1 % formic acid in MeOH and 0.1% formic acid as solvents) $^1H$ NMR (400 MHz, $CDCl_3$) δ 10.67 (s, 1H), 7.85-7.75 (m, 1H), 7.60-7.42 (m, 5 H), 7.27-7.25 (m, 2H), 5.10 (s, 1H), 2.69 (s, 3H), 2.64 (s, 3H), 1.03 (s, 9H). Aromatic regions in $^1H$ NMR (400 MHz, DMSO) δ 7.76 (s, 1H), 7.72 (d, *J* = 8 Hz, 1H), 7.63 - 7.46 (m, 3H), 7.33 - 7.1(m, 3H). HRMS-ESI *m/z* $(M+H)^+$ calcd for $C_{26}H_{26}ClN_3O_3$, 464.1741 found 464.1726.
